# Withholding tax rates on dividends: symmetries versus asymmetries or single- versus multi-rated double tax treaties

**DOI:** 10.1007/s10797-020-09637-y

**Published:** 2020-12-20

**Authors:** Kunka Petkova

**Affiliations:** Doctoral Program in International Business Taxation (DIBT), Vienna University of Economics and Business, 1020 Vienna, Austria

**Keywords:** Double tax treaties, Withholding tax rates, Dividends, Portfolio dividends, Participation dividends, F23, F53, H25, H26, H73, H87, K34

## Abstract

Out of all double tax treaties (DTTs) in force in 2012, around 41% are symmetric (single-rated) and 59% are asymmetric (multi-rated), i.e., they prescribe different dividend withholding tax rates depending on the foreign investor’s ownership fraction. The paper investigates the reasons for this phenomenon, namely why some countries in their DTTs prefer homogenous withholding tax rates over separate rates for participation and portfolio dividends. In a theoretical model, I demonstrate why home countries may have an interest in a high withholding tax rate in the host country, even though they do not receive the revenue from this tax. Further, I find confirming evidence that a reason for having multi-rated withholding taxes on dividends is an existing spatial dependence on the rates of the countries’ peers that may be a driving factor for setting multi-rated taxes. Finally, I confirm that the spread itself (i.e., the difference between the portfolio and participation dividends negotiated in the tax treaty) is also affected by the peer countries.

## Introduction

Double taxation or the levying of tax by two jurisdictions on the same declared income, asset or transaction is not a recent problem. Even though residence countries usually provide some method of double tax relief in an attempt to prevent double taxation unilaterally, for instance, by giving the investors a credit for tax paid abroad or by exempting foreign-source income from domestic tax, these attempts are mostly imperfect. Therefore, with the expansion of transportation and the fast-growing rates of capital transactions, double tax treaties have started to emerge. Their main role is to set out rules to avoid double taxation.

Prior literature on double tax treaties (DTTs) primarily focuses on their effects on foreign direct investment (FDI) or on the formation side of DTTs (see Van’t Riet and Lejour 2018; Hong 2018; Chisik and Davies 2004). Accounting for the opportunities for treaty shopping, Petkova et al. (2020a) show that relevant tax treaties—which reduce repatriation taxes on dividends both over domestic law and the entire existing treaty network—will increase FDI by about 18%. Ligthart et al. (2011) use a gravity model to conclude that countries sign DTTs mainly to reduce international double taxation and, to a lesser extent, to provide a legal instrument for the exchange of information in tax matters. Despite these contributions, certain parts of the international tax treaty policy still remain unexplored. In this paper, I concentrate on the results for withholding tax rates (WTRs), in particular those for dividends.

Generally, cross-border portfolio investments trigger withholding taxes in the source countries, i.e., the countries in which the funds are invested. Applying withholding taxes may be justified by the fact that foreign investors would otherwise benefit from the infrastructure of the source country without contributing enough to it by just paying the corporate income taxes (Taxology 2018). Therefore, WTRs are often levied to ensure the collection of taxes, especially in situations in which the income would possibly escape taxation (Willis 1963). Also, withholding taxes are a simple way of administering taxes, in particular because non-residents are less available to the tax authorities than residents. The most common withholding tax rates are those on dividends, interest and royalties. They are called withholding taxes because, even though the foreign investor is the taxpayer, they are withheld from the dividends or interest paid by the company in which the foreign investor has invested and remitted to the source country’s tax administration (Taxology 2018). In other words, they are to be paid to the tax administration by the payer rather than the recipient.

When it comes to dividends, there is an important distinction as often one of two possible rates in the form of portfolio and participation dividends may apply. In particular, out of all double tax treaties in force in 2012, around 41% are symmetric (single-rated) and 59% are asymmetric (multi-rated), i.e., they prescribe different dividend withholding tax rates depending on the foreign investor’s ownership fraction. Often, companies owning less than a specified percentage of shares in a foreign company are granted only a limited reduction in the standard rate of withholding tax. Such shareholders are known as portfolio shareholders (Deloitte International Tax Source 2020). Shareholders owning more than the prescribed limit are often granted a more generous reduction or even elimination of withholding tax and are labeled significant shareholders.

To my knowledge, this is the first, and so far only, paper dealing with this phenomenon, namely why some countries in their double tax conventions prefer homogenous withholding tax rates over separate rates for participation and portfolio dividends. Such differentiation is typically absent under national law. This paper investigates the reasons for the asymmetry in the withholding tax rates in DTTs. One possible explanation for the higher withholding tax rate on portfolio dividends is that tax avoidance in the case of portfolio dividends is more likely. In a theoretical model presented in Appendix 1, I demonstrate why home countries may have an interest in a high withholding tax rate in the host country, even though they do not receive the tax revenue from this tax. This may happen since the withholding tax rate abroad helps them to decrease domestic tax avoidance and increase thereby the tax revenue in the home country. In Sect. 4.1, I test this hypothesis with the existent data and present confirming evidence.

Further, one hypothesis for having multi-rated withholding taxes on dividends is an existing spatial dependence on the rates of the countries’ peers that may be a driving factor for setting split rates. I confirm this hypothesis in Sect. 4.2.

In the remainder of the paper, I provide more information on the dataset and some summary statistics in Sect. 2. In Sect. 3, I look at the development of the withholding tax rates on dividends and their international tax competition over time. Section 4 investigates possible reasons for the differentiation between the withholding tax rates, and the hypotheses are tested empirically. Finally, Sect. 5 concludes.

## Data and descriptive statistics

**Fig. 1:**
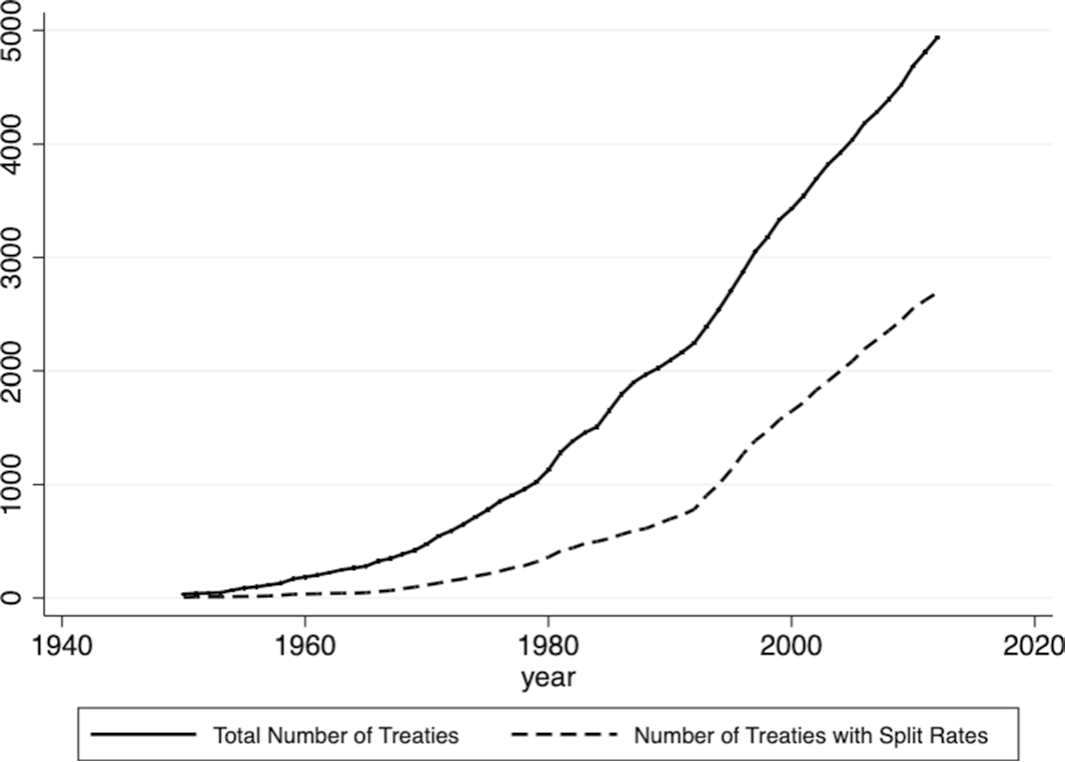
Number of treaties and treaty types over the years

The dataset covers 131 countries and 2470 double tax treaties signed between 1950 and 2012 (see Fig. 1). This implies that of all possible country pairs in the sample, 29% actually have a double tax treaty in force in 2012. Further, the treaty network might be subject to changes, such as effective new treaties in place, termination of treaties and changes due to subsequent protocols. Therefore, I take all of those into account and update the dataset accordingly, while going through every single–double tax treaty and manually collecting the data on the WTRs.

Table 1 summarizes the possible constellations for the withholding tax rates on dividends of the treaty partners and their corresponding number in the sample. The first one (Column 1: Uniform rates) consists of uniform and equal rates for both countries. This means that the two treaty partners have only one withholding tax rate on dividends and it is the same in both directions. A further option is that each of the two treaty partners has again only one withholding tax rate on dividends, but this one is not the same in both directions. What is more, countries might have split rates on dividends (Column 2: Split rates both countries). Four scenarios are possible: They have equal rates on both dividend types; they have equal rates only on the lower rate on participation dividends; they have equal rates only on the higher rate on portfolio dividends; or they do not have any equal rates. Finally, it is possible that only one of the treaty partners has split rates in the double tax treaty (Column 3: Split rates one country). Here, three further subcases are feasible.^1^Overall, it can be seen from Table 1 that out of all double tax treaties in the sample 41% are single-rated (Column 1: Uniform rates) and 59% are multi-rated (Column 2: Split rates both countries; Row 1: Equal rates both rates), whose development is shown in Fig. 1. In the remainder of the paper, I will look at those cases without any further differentiation.

**Table 1:**
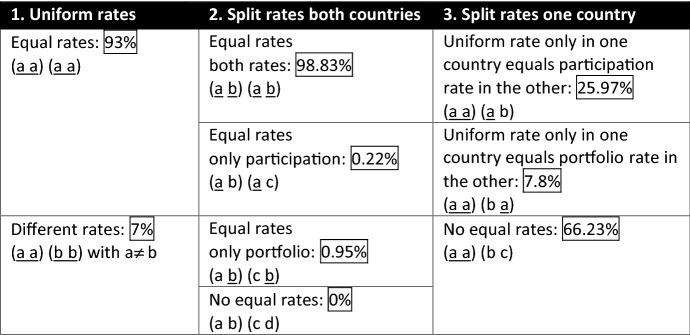
Constellations for the withholding tax rates on dividends in the double tax treaties in 2012

Note that the first parenthetic term (portfolio, participation) represents the two withholding tax rates on dividends of the first treaty partner that could be equal or different from each other. The second parenthetic term depicts correspondingly the two withholding tax rates on dividends of the second treaty partner that could again be equal or different from each other

## Withholding tax rates on dividends and international tax competition

Over the last decades, barriers to capital movement have become significantly lower. As a result of this, capital is expected to move where taxes are lowest. Standard contributions to the tax competition literature predict that the increased capital mobility will tend to erase source-based taxes on mobile capital. Most empirical studies focus on the corporate business tax as an important source-based tax on capital. Indeed, statutory corporate tax rates in developed countries have fallen substantially over the last two decades. The average rate among OECD countries in the early 1980s was nearly 50% (OECD Tax Database), and by 2001 it had fallen to under 35%. Further, average statutory corporate tax rates around the world have declined from 32.2% in 2000 to 24.7% in 2016 (Hannon 2017). Devereux, Lockwood and Redoano (2008) find evidence that individual countries cut their own corporate tax rate as a reaction to cuts in the average tax rates in other countries.

However, the corporate income tax is not the only source-based tax on capital—source-based taxes are also applied in the form of withholding taxes on dividends. The purpose of this section of the paper is to fill a gap in the literature by asking why and to what extent tax rates of cross-border flows such as dividends have survived over the years. With some qualifications applying, the international tax competition models would predict zero, or at least declining rates.

**Fig. 2:**
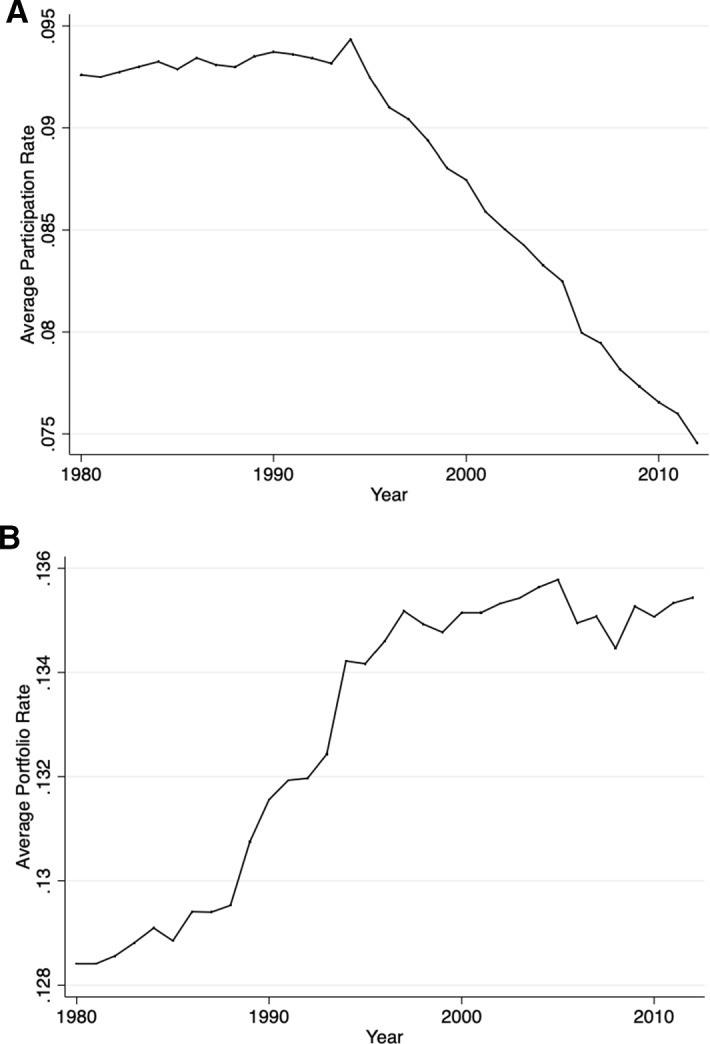
**a** Average WTRs on participation dividends if directed country pair existed 1980. **b** Average WTRs on portfolio dividends if directed country pair existed 1980

**Fig. 3:**
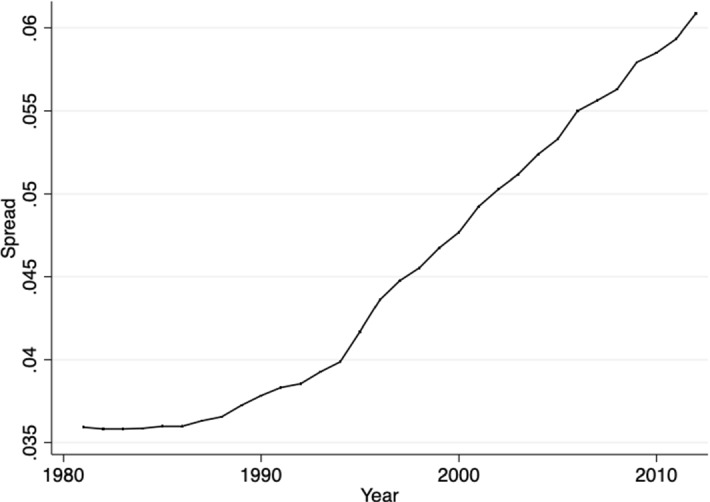
Development of the average spread between WTRs on portfolio and participation dividends over time if directed country-pair existed 1980

**Fig. 4:**
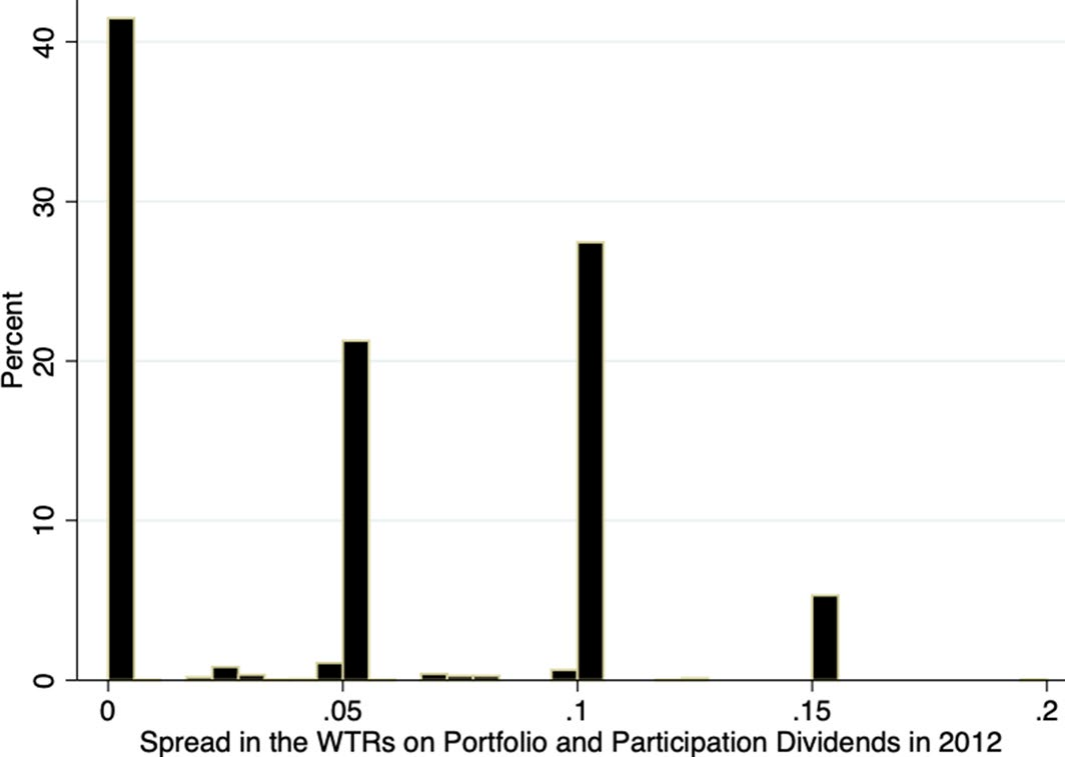
Distribution of the spread between WTRs on portfolio and participation dividends in 2012

The current section presents the development of the average withholding tax rates on participation and portfolio dividends over time. For the purpose of this exercise, I look at the existing country pairs that had a double tax treaty in 1980 and keep them fixed, so that I can observe all changes that happened between 1980 and 2012.^2^ The reason for choosing 1980 as the starting year is the fact that the international tax competition^3^intensified thereafter and more than 30% of the signed double tax treaties were already in force. The changes in the averages of the withholding tax rates on dividends may be driven by a new effective double tax treaty signed between the country-pair members, by a termination of the existing treaty or by changes via subsequent protocols and subsequent treaties with increases or decreases in the corresponding tax rates. Figure 2a, b depicts this development. Figure 3 shows the development of the average spread between WTRs on portfolio and participation dividends after 1980, and Fig. 4 depicts its distribution for the last year of the sample.

Interestingly, the trends differ for the WTR on participation and portfolio dividends. While in the case of participation dividends a downward trend after 1990 may be observed, portfolio dividends are characterized by an upward trend in the years before mid-1990 and remain relatively stable after that. Since the early 1980s, tax treaty WTRs on portfolio dividends for the directed country pairs that existed in the year 1980 have on average increased by about 5.5% (or 0.7% points), while the average rate on participating dividends has fallen almost by 19.5% (or 2% points) until 2012. If the development is due to competitive forces, then this will suggest that the international tax competition is stronger when it comes to participation dividends.

When it comes to the spread between the withholding tax rates on portfolio and participation dividends, Fig. 3 shows that the average spread increased over time and almost doubled in the period between 1980 and 2012, which is in line with the development presented in Fig. 2a, b. Some countries that previously did not differentiate between the two types of WTRs introduced different rates for portfolio and participation dividends.

**Fig. 5:**
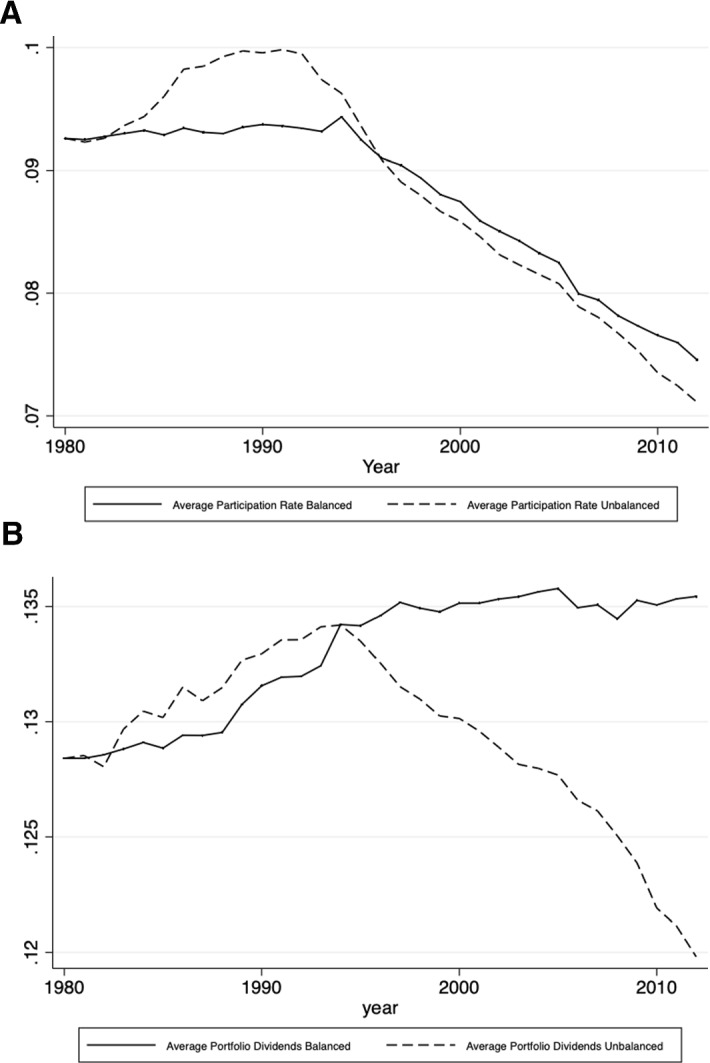
**a** Average WTRs on participation dividends with unbalanced sample. **b** Average WTRs on portfolio dividends with unbalanced sample

Finally, Fig. 5a, b shows the change in the average withholding tax rates on participation and portfolio dividends for the same period. However, unlike in the previous figures, here the unbalanced average among all double tax treaties (and not only those of the existing country pairs in 1980) in the particular year is presented. What can be seen is that participation dividends are again characterized by a more dynamic development than the one of portfolio dividends and face a steeper decrease after 1990. In the case of portfolio dividends, there is also a decrease in the average rates over time, despite being very small. Nevertheless, the results in Fig. 5 may be due to a possible composition effect, as many new treaties have been signed over the period between 1980 and 2012.^4^

Overall, it can be seen from all graphs that in line with the international tax competition models, declining withholding tax rates on dividends are observed when it comes to participation dividends, while the prediction of declining rates is less visible in the case of portfolio dividends. The remainder of Sect. 3 presents three possible explanations for the differences in the international tax competition in the withholding tax rates on participation and portfolio dividends.

One possible explanation for these differences may be connected to treaty shopping. Treaty shopping is the practice of taking advantage of the international tax treaty network and the most favorable tax treaty. For instance, this is the case if a person resident of a given State (State R) expects to derive dividends sourced in another State (State S) and decides to set up an entity in a third state (State C) that will receive the dividends in a more beneficial way than if such income were paid directly from State S to the person resident of State R (IBFD 2008). The reason for the tax advantage lies in the fact that the tax treaty between State S and State C provides for a lower withholding tax rate in State S on dividends paid to a State C resident than the rate that would apply in State S if the income were paid directly to the State R resident. This occurs because there is either no treaty applicable between State R and State S or, if there is one, it provides for less generous withholding tax rates than those available to the State C resident under the treaty between S and C (IBFD 2008). Due to the fact that such treaty shopping is not possible for portfolio dividends, unlike in the case of participation dividends, there is no incentive to set the tax rates lower. Thus, the international tax competition may be stronger when it comes to participation dividends.

A possible argument for positive withholding tax rates on dividends can be found in Gordon (1992) who discusses whether capital income tax can survive in an open economy. Thinking of the USA as a Stackelberg leader, he argues that the rationale for the USA giving tax credits (until 2017) may have been that this encouraged other countries to impose source-based capital income tax and, by doing so, prevented a capital flight that may otherwise erode the domestic capital income tax base. Moreover, as long as the withholding tax rate remains below the rate under domestic law faced by foreign investors on their portfolio income, the tax produces revenue without any loss to domestic residents. Therefore, for the countries offering a tax credit as their method of double tax relief, the rationale for including positive WTRs in the double tax treaties may be explained.

Another explanation for the increasing international tax competition in the case of participation dividends may be the rising pressure coming from the fact that the number of countries adopting the territorial tax system is rising (e.g., the UK and Japan in 2009). In a pure territorial tax system, the country taxes only corporations’ income derived within its borders. This is normally achieved by exempting from the domestic tax base the dividends received from foreign subsidiaries. By contrast, in a pure worldwide tax system, resident corporations are taxable on their worldwide income regardless of where the income is derived. For example, before the change in the tax system in 2018, a US investor directly investing in a country that has withholding tax rates can still be taxed on a residence basis even if he lives in a country that has a territorial tax system. Therefore, if the majority of the inbound investment were from countries with a worldwide tax system, withholding taxes in the source countries would have had lesser impact, and therefore tax competition would be cushioned. At the same time, there is no move to territorial taxation for individuals, i.e., dividends received by individuals are usually taxable even in exemption countries. So, to the extent that portfolio dividends are paid to individuals, for these dividends a tax credit in the home country of the investor may be available and higher WTRs may not shy away these investors. This would help explain why there is a lesser response on the portfolio dividends, as shown in Fig. 2b.

## Possible reasons for split withholding tax rates on dividends

In Sect. 4, I investigate the reasons for split rates in the withholding tax rates on dividends. The section consists of two subsections. Section 4.1 shows why home countries may have an interest in a high withholding tax rate in the host country or why they adopt multi-rated tax treaties. Section 4.2 shows whether and how multi-rated tax treaties are disseminated through neighboring countries’ existing treaties.

### Why may home countries have an interest in a high withholding tax rate in the host country or why do they adopt multi-rated tax treaties?

According to the International Monetary Fund (2010), there are two subgroups of foreign equity investments: foreign portfolio investments (FPI) and foreign direct investments (FDI). The foreign equity investments are defined as FDI (FPI) if investments contain more (less) than 10% of the controlling rights. The source country’s withholding tax rates applicable to dividends from FDI are typically lower than the rates for FPI—when targeted to specific countries via double tax treaties. If the minimum equity participation is not met, the portfolio withholding tax rates on the dividends will apply.

The ways in which countries mitigate corporate taxation on dividend income are also different depending on whether this income is from FDI or FPI. For instance, with minimum equity participations being at least 10% of the foreign corporation, residence countries with a worldwide tax system provide a tax credit to a resident corporate shareholder for corporate income taxes paid by the foreign corporation (i.e., an indirect tax credit). Other countries mitigate corporate taxation on dividend income from FDI through dividends exemption or the use of a territorial tax system by taxing only domestic income. When it comes to foreign portfolio investments, most residence countries provide a tax credit for withholding taxes paid to the source country.

In the theoretical model in Appendix 1, I use the feature that portfolio investors are usually taxed using the credit system and find why the home country may have an interest in a high withholding tax rate on dividends in the host country. If lying about the declared income at home gets costlier (for example, via exchange of information), the withholding tax rate of the host country is less relevant for the home country, as the honesty of the investor has increased. Overall, I find that there is a negative connection between the tax avoidance in the home country and the withholding tax rate of the partner country.

The remainder of the section will test whether for countries, which may be worried about tax avoidance and tax evasion and have a high number of concluded Tax Information Exchange Agreements (TIEAs), such an interest in a high tax treaty withholding rate on portfolio dividends in the host country is reflected in tax treaties. As laid out above, a high withholding tax rate on portfolio dividends increases investor’s honesty and thereby his declared income at home, which may lead to more tax revenue in the home country (see Appendix 1, Eq. (3) of the theory model). Therefore, the hypothesis is that the extent to which countries are worried about tax evasion and tax avoidance will result in a high withholding tax rate on portfolio dividends in the host country and will not affect the withholding tax rate on participation dividends. The idea is that tax avoidance and tax evasion are likely for participation dividends and may occur via treaty shopping. However, as this option is more difficult for portfolio dividends and the high WTRs cannot be avoided so easily, countries may still have an incentive to include high WTR on portfolio dividends in their double tax treaties if they are worried about tax avoidance and tax evasion.

For the empirics, I use a pooled cross section across all years with available domestic tax data^5^ (between 1950 and 2012) and an OLS estimation methodology with time-, home- and host-fixed effects:

$${\text{WTR}}_{j,t} = \alpha + \beta_{i} X_{i,t} + \beta_{j} X_{j,t} + \theta_{i} + \vartheta_{j} + \lambda_{t} + \xi_{ij,t} .$$

The dependent variable (${\text{WTR}}_{j,t}$) is the tax treaty withholding rate on portfolio dividend in the host country *j* in Table 2 and the tax treaty withholding rate on participation dividend in the host country *j* in Table 3.$X_{i,t}$ and $X_{j,t}$ are the vectors of the control variables. Among those explanatory variables are the dummies taking the value of unity if home (host) country is an OECD member, *OECD_o* (*OECD_d*), as well as *OECD_pair* for the cases in which both home and host countries are OECD members. There are also dummy variables taking the value of unity if home (host) country is a EU member, *EU_o* (*EU_d)*, as well as *EU_pair* for the cases in which both home and host countries are EU members. The same logic applies for the tax haven dummies: *taxhaven_o*, *taxhaven_d* and *taxhaven_pair*. Further, the variables *taxdividends_o* and *taxdividends_d* capture the domestic withholding tax on dividends (i.e., the withholding tax that applies where final shareholder-level tax is withheld by the distributing company), respectively, in home and host country. There are also control variables depicting GDP (*gdp_o* and *gdp_d*), as well as GDP per capita (*gdpcap_o* and *gdpcap_d*) in both countries. Moreover, the variables *tieas_o* and *tieas_d* account for the number of TIEAs that home and host countries have in the particular year. These two variables are proxies^6^ for the extent to which non-haven countries are worried about tax avoidance and tax evasion. The rationale is that the more the TIEAs there are in place, the higher the extent is to which countries are concerned about their residents trying to reduce the tax liability. Finally, $\theta_{i}$ and $\vartheta_{j}$ are home-, respectively, host-fixed effects; $\lambda_{t}$ is a vector of year dummies, and $\xi_{ij,t}$ is the error term.

**Table 2:** Tax treaty withholding rates on portfolio dividends in the host country

Variables	(1) WTR on host’ portfolio dividends	(2) WTR on host’ portfolio dividends	(3) WTR on host’ portfolio dividends	(4) WTR on host’ portfolio dividends
tieas_o		8.73e−05***		0.000103***
		(3.36e−05)		(3.45e−05)
tieas_d		8.78e−05**		0.000101**
		(3.89e−05)		(4.03e−05)
gdp_o	− 8.04e−17	2.80e−17		
	(2.34e−16)	(2.36e−16)		
gdp_d	5.05e−16*	6.11e−16**		
	(2.87e−16)	(2.89e−16)		
gdpcap_o	8.09e−08**	6.85e−08*		
	(3.82e−08)	(3.86e−08)		
gdpcap_d	5.76e−08	4.58e−08		
	(4.18e−08)	(4.19e−08)		
ln_gdp_o			− 0.00187	− 0.000774
			(0.00517)	(0.00514)
ln_gdp_d			− 0.00779	− 0.00670
			(0.00508)	(0.00506)
ln_gdpcap_o			0.00248	0.00242
			(0.00534)	(0.00533)
ln_gdpcap_d			0.00899*	0.00893*
			(0.00526)	(0.00525)
OECD_o	− 0.00393	− 0.00357	− 0.00396	− 0.00347
	(0.00242)	(0.00242)	(0.00242)	(0.00242)
OECD_d	− 0.00245	− 0.00210	− 0.00257	− 0.00210
	(0.00234)	(0.00234)	(0.00233)	(0.00234)
OECD_pair	− 0.00396	− 0.00399	− 0.00396	− 0.00400
	(0.00296)	(0.00296)	(0.00296)	(0.00296)
EU_o	− 0.00480**	− 0.00442**	− 0.00536**	− 0.00503**
	(0.00207)	(0.00208)	(0.00210)	(0.00210)
EU_d	− 0.00445**	− 0.00410**	− 0.00560***	− 0.00530**
	(0.00206)	(0.00207)	(0.00209)	(0.00209)
EU_pair	0.00411	0.00414	0.00411	0.00413
	(0.00321)	(0.00321)	(0.00321)	(0.00321)
taxhaven_o	− 0.00657	− 0.00671	− 0.00638	− 0.00783
	(0.0133)	(0.0133)	(0.0133)	(0.0134)
taxhaven_d	− 0.0110	− 0.0112	− 0.0107	− 0.0122
	(0.0146)	(0.0146)	(0.0147)	(0.0147)
taxhaven_pair	− 0.00607	− 0.00607	− 0.00605	− 0.00605
	(0.00654)	(0.00654)	(0.00654)	(0.00654)
taxdividends_o	− 0.00270	− 0.00159	− 0.00308	− 0.00147
	(0.00373)	(0.00381)	(0.00367)	(0.00376)
taxdividends_d	0.00272	0.00377	0.00228	0.00380
	(0.00328)	(0.00339)	(0.00323)	(0.00338)
Time-fixed effects	Yes	Yes	Yes	Yes
Home-fixed effects	Yes	Yes	Yes	Yes
Host-fixed effects	Yes	Yes	Yes	Yes
Observations	33,310	33,310	33,310	33,310
R-squared	0.468	0.468	0.468	0.468

Robust standard errors in parentheses are clustered by country-pair ***p < 0.01; **p < 0.05; *p < 0.1

The dependent variable is the tax treaty withholding rate on portfolio dividends in the host countries

**Table 3:** Tax treaty withholding rates on participation dividends in the host country

Variables	(1) WTR on host’ participation dividends	(2) WTR on host’ participation dividends	(3) WTR on host’ participation dividends	(4) WTR on host’ participation dividends
tieas_o		− 3.95e−05		− 2.35e−05
		(3.25e−05)		(3.52e−05)
tieas_d		− 4.17e−05		− 2.93e−05
		(3.77e−05)		(3.96e−05)
gdp_o	− 6.42e−16*	− 6.91e−16**		
	(3.37e−16)	(3.40e−16)		
gdp_d	− 1.93e−16	− 2.43e−16		
	(3.38e−16)	(3.40e−16)		
gdpcap_o	− 9.79e−08**	− 9.23e−08**		
	(4.07e−08)	(3.98e−08)		
gdpcap_d	− 1.25e−07***	− 1.19e−07**		
	(4.69e−08)	(4.66e−08)		
ln_gdp_o			0.00705*	0.00680*
			(0.00367)	(0.00368)
ln_gdp_d			0.00393	0.00362
			(0.00370)	(0.00370)
ln_gdpcap_o			− 0.00507	− 0.00505
			(0.00374)	(0.00374)
ln_gdpcap_d			− 0.00158	− 0.00157
			(0.00382)	(0.00381)
OECD_o	0.00191	0.00175	0.00224	0.00213
	(0.00261)	(0.00261)	(0.00262)	(0.00263)
OECD_d	0.00427*	0.00410*	0.00450*	0.00436*
	(0.00230)	(0.00231)	(0.00231)	(0.00232)
OECD_pair	− 0.00682**	− 0.00680**	− 0.00680**	− 0.00679**
	(0.00334)	(0.00334)	(0.00334)	(0.00334)
EU_o	− 0.000201	− 0.000372	0.000393	0.000315
	(0.00223)	(0.00223)	(0.00225)	(0.00225)
EU_d	− 1.83e−05	− 0.000185	0.000263	0.000181
	(0.00223)	(0.00223)	(0.00225)	(0.00225)
EU_pair	− 0.00101	− 0.00102	− 0.00106	− 0.00106
	(0.00369)	(0.00369)	(0.00369)	(0.00369)
taxhaven_o	0.0137	0.0137	0.0101	0.0104
	(0.0176)	(0.0176)	(0.0178)	(0.0178)
taxhaven_d	0.0125	0.0126	0.00885	0.00927
	(0.0190)	(0.0190)	(0.0192)	(0.0192)
taxhaven_pair	− 0.00124	− 0.00123	− 0.00124	− 0.00124
	(0.00498)	(0.00498)	(0.00498)	(0.00498)
taxdividends_o	− 0.00889**	− 0.00939**	− 0.00741*	− 0.00778**
	(0.00383)	(0.00387)	(0.00378)	(0.00377)
taxdividends_d	− 0.00251	− 0.00301	− 0.000948	− 0.00138
	(0.00256)	(0.00265)	(0.00254)	(0.00263)
Time-fixed effects	Yes	Yes	Yes	Yes
Home-fixed effects	Yes	Yes	Yes	Yes
Host-fixed effects	Yes	Yes	Yes	Yes
Observations	33,310	33,310	33,310	33,310
R-squared	0.453	0.453	0.453	0.453

Robust standard errors in parentheses are clustered by country-pair ***p < 0.01; **p < 0.05; * p < 0.1

The dependent variable is the tax treaty withholding rate on participation dividends in the host countries

Tables 2 and 3 present the results, and Table 9 in the appendix shows the summary statistics for the estimated sample. The coefficient of TIEAs in the home country (*tieas_o*) on the withholding tax rates on portfolio dividends in the host country is statistically significant and positive in all columns and corresponds to the presented theory: Countries that seem worried about tax avoidance and tax evasion and have a high number of concluded TIEAs may have an interest in a high tax treaty withholding rate on portfolio in the host country, as the high WTR increases investor’s honesty and thus his declared income and the tax revenue in the home country. At the same time, the coefficient of the concluded TIEAs in the host country is positive, indicating that those also seem to matter for the size of the withholding tax rates on portfolio dividends in the host country. Further, the coefficients of the EU countries dummies are negative and statistically significant across all columns in Table 2. This implies that EU countries have lower withholding tax rates on portfolio dividends in the host country.

Fears about tax evasion refer to portfolio dividends, but are usually absent for dividends paid within multinational firms. Indeed, here dividends are often exempted and there is no incentive for underreporting in the home country. When it comes to the withholding tax rates on participation dividends, Table 3 indicates that the proxies for tax avoidance and tax evasion are not statistically significant. The finding implies that there is no connection between the extent to which countries are worried about tax avoidance and tax evasion and the withholding tax rate on participation dividends. Hence, this insignificance may be interpreted as a successful placebo test. Interestingly, if both countries are OECD members, they are more likely to negotiate in their double tax treaties a lower WTR on participation dividends.

Finally, Table 4 reports a regression in which the dependent variable is the spread between the withholding tax rates on portfolio and participation dividends. It can be seen that if countries are worried about tax avoidance and tax evasion (i.e., they have a high number of TIEAs), they are more likely to negotiate a high spread between the withholding tax rates in the portfolio and participation dividends in their double tax treaties. The high spread may result from a higher WTR on portfolio dividends or a lower WTR on participation dividends. However, from Tables 2 and 3, we know that only the effect of TIEAs on the WTR on portfolio dividends is statistically significant. Therefore, countries that are more worried about tax avoidance and tax evasion are also more likely to have a higher WTR on portfolio dividends in the host country and have thereby a higher spread between the WTRs on portfolio and participation dividends. What is more, OECD and EU countries have a lower spread between the WTRs on dividends. This may be due to lower WTR on portfolio or higher WTR on participation dividends. Finally, if there is a high taxation on dividends in domestic law of home/host country, the spread between the WTRs on portfolio and participation dividends is higher as well.

**Table 4:** Spread between the WTRs on portfolio and participation dividends in the host country

Variables	(1) Spread on partner’s dividends	(2) Spread on partner’s dividends	(3) Spread on partner’s dividends	(4) Spread on partner’s dividends
tieas_o		0.000127***		0.000127***
		(3.52e−05)		(3.67e−05)
tieas_d		0.000130***		0.000130***
		(3.76e−05)		(3.91e−05)
gdp_o	5.62e−16*	7.19e−16**		
	(3.39e−16)	(3.36e−16)		
gdp_d	6.99e−16**	8.54e−16**		
	(3.52e−16)	(3.50e−16)		
gdpcap_o	1.79e−07***	1.61e−07***		
	(4.27e−08)	(4.18e−08)		
gdpcap_d	1.83e−07***	1.65e−07***		
	(4.32e−08)	(4.25e−08)		
ln_gdp_o			− 0.00892**	− 0.00757*
			(0.00411)	(0.00409)
ln_gdp_d			− 0.0117***	− 0.0103**
			(0.00404)	(0.00402)
ln_gdpcap_o			0.00755*	0.00747*
			(0.00426)	(0.00424)
ln_gdpcap_d			0.0106**	0.0105**
			(0.00419)	(0.00417)
OECD_o	− 0.00584**	− 0.00532**	− 0.00620***	− 0.00560**
	(0.00236)	(0.00236)	(0.00237)	(0.00236)
OECD_d	− 0.00672***	− 0.00620***	− 0.00707***	− 0.00647***
	(0.00229)	(0.00229)	(0.00229)	(0.00230)
OECD_pair	0.00286	0.00281	0.00284	0.00279
	(0.00329)	(0.00330)	(0.00329)	(0.00330)
EU_o	− 0.00460**	− 0.00405*	− 0.00575***	− 0.00535**
	(0.00214)	(0.00215)	(0.00215)	(0.00215)
EU_d	− 0.00443**	− 0.00391*	− 0.00586***	− 0.00549***
	(0.00212)	(0.00213)	(0.00213)	(0.00213)
EU_pair	0.00511	0.00516	0.00517	0.00520
	(0.00373)	(0.00373)	(0.00373)	(0.00373)
taxhaven_o	− 0.0202	− 0.0204	− 0.0164	− 0.0182
	(0.0131)	(0.0131)	(0.0133)	(0.0132)
taxhaven_d	− 0.0236*	− 0.0238*	− 0.0196	− 0.0214*
	(0.0128)	(0.0128)	(0.0129)	(0.0129)
taxhaven_pair	− 0.00483	− 0.00483	− 0.00482	− 0.00481
	(0.00526)	(0.00527)	(0.00527)	(0.00527)
taxdividends_o	0.00619**	0.00780***	0.00434	0.00631**
	(0.00282)	(0.00297)	(0.00280)	(0.00295)
taxdividends_d	0.00524*	0.00678**	0.00323	0.00518*
	(0.00285)	(0.00300)	(0.00281)	(0.00298)
Time-fixed effects	Yes	Yes	Yes	Yes
Home-fixed effects	Yes	Yes	Yes	Yes
Host-fixed effects	Yes	Yes	Yes	Yes
Observations	33,310	33,310	33,310	33,310
R-squared	0.413	0.413	0.413	0.413

Robust standard errors in parentheses are clustered by country-pair ***p < 0.01; **p < 0.05; *p < 0.1

The dependent variable is the spread between the tax treaty WTR on portfolio and participation dividends in the host countries

The above-mentioned findings may be the explanation of Fig. 2A and 2B in the paper. From Sect. 2, we know that the WTRs on portfolio dividends are characterized by an upward trend in the years before mid-1990 and remain relatively stable after that (see Fig. 2a). One explanation for this may be found in the current empirical section and may be related to the increasing concern of tax avoidance and tax evasion. Countries that are more worried about tax avoidance and tax evasion are more likely to negotiate a higher treaty WTR on portfolio dividends in the host country. At the same time, there is a downward trend of the WTR on participation dividends over the years (see Fig. 2b). As tax avoidance and tax evasion are likely for this type of dividends due to treaty shopping, countries may not try to keep them high and tax competition may dominate.

The increasing spread between the WTRs of the portfolio and participation dividends may be an indicator that some countries are increasingly worried about tax avoidance and tax evasion in the case of portfolio dividends but are willing to reduce rates for participation dividends where tax avoidance is a much lesser issue.

### Do multi-rated tax treaties of peer countries let other countries take such treaties?

Previous studies on withholding tax rates suggest a spatial dependence between countries’ policies. Section 4.2 examines whether this holds for the decision on split rates for portfolio and participation dividends and whether those are affected by the peer countries. Chisik and Davies (2004) and Barthel and Neumayer (2012) propose that countries consider the spatial interdependencies in the global tax treaties network when negotiating their treaty rates. They show that the probability of two countries concluding a DTT increases with peer countries having signed a treaty with the same treaty partner. However, their findings are limited to the diffusion of DTTs as such and do not consider their content. Petkova et al. (2020b) fill this void by extending the tax treaty bargaining framework to spatial dependence in treaty withholding tax rates. The authors show that tax treaty rates are influenced by treaty rates negotiated by any of the two signatory countries with the peers of the other one and find a positive relationship between the spatial interaction terms and the negotiated withholding tax rates. Following up on this finding, the current paper wants to see whether there are spillover effects^7^ also when it comes to multi-rated withholding taxes on dividends, i.e., the decision to have split rates. Therefore, the hypothesis to be tested is that countries are influenced by the already signed tax treaties and the withholding tax rates negotiated by their peers, when deciding on having split withholding tax rates in their double tax treaties. The second hypothesis to be tested is whether the spread itself (i.e., the difference between the WTRs on portfolio and participation dividends negotiated in the tax treaty) is also affected by the peer countries.

For the purpose of testing both hypotheses, I estimate a pooled cross section across all years in the sample in the following form (standard errors are clustered by country-pair) using OLS with multiple high-dimensional fixed effects (HDFE), so that I can control for multiple sources of heterogeneity:

$${\text{Split}}_{ij,t} = \alpha + \rho_{j} {\text{Split}}_{j,t - n} \omega_{j} + \beta_{i} X_{i,t} + \beta_{j} X_{j,t} + \gamma V_{ij,t} + \theta_{i} + \vartheta_{j} + \lambda_{t} + \varsigma r_{i,t} + \chi r_{j,t} + \xi_{ij,t}$$

$${\text{Spread}}_{ij,t} = \alpha + \rho_{j} {\text{Spread}}_{j,t - n} \omega_{j} + \beta_{i} X_{i,t} + \beta_{j} X_{j,t} + \gamma V_{ij,t} + \theta_{i} + \vartheta_{j} + \lambda_{t} + \varsigma r_{i,t} + \chi r_{j,t} + \xi_{ij,t}$$

where ${\text{Split}}_{ij,t}$ is an indicator variable taking the value of one if there is an asymmetry (i.e., two different rates) in the treaty withholding tax rates on dividends between source country *i* and target country *j* in year of treaty conclusion *t*; $\rho_{j} {\text{Split}}_{j,t - n} \omega_{j}$ is the spatial interaction term between the spatial weight matrix at a subregion level of the target country *j* and an asymmetry withholding tax rates matrix of source country *i* with all other potential targets *m*, *n*^8^years before treaty year—target lag.^9^ Analogically, for the second estimation, the dependent variable ${\text{Spread}}_{ij,t}$ is the difference in the WTRs on portfolio and participation dividends in the tax treaty between source country *i* and target country *j* in year of treaty conclusion *t*; $\rho_{j} {\text{Spread}}_{j,t - n} \omega_{j}$ is the spatial interaction term between the spatial weight matrix at a subregion level of the target country *j* and a spread withholding tax rates matrix of source country *i* with all other potential targets *m*,*n* years before treaty year—target lag. Further, $X_{i,t}$ and $X_{j,t}$ are vectors of source, respectively, target-specific factors that affect their bargaining position, especially GDP and GDP per capita; *V* is a vector of variables characterizing the bilateral relationship between source *i* and target *j*; $\theta_{i}$ and $\vartheta_{j}$ are source, respectively, target-region-country fixed effects; $\lambda_{t}$ is a vector of year dummies; $\varsigma r_{i,t}$ and $\chi r_{j,t}$ are source-region-year, respectively, target-region-year fixed effects; and $\xi_{ij,t}$ is the error term. The independent variables are the same as in Tables 2, 3 and 4. Further, I alleviate the concerns about the endogeneity of the spatial interaction terms by exploiting the time dimension of tax treaty bargaining. In particular, I lag the spatial interaction terms 2 years before treaty conclusion in year *t*—i.e., before the corresponding withholding tax rates and the existence of a split in the dividend rates are being observed—and assume that while past treaty rates can affect the yet-to-be-negotiated ones, this relationship does not reverse. Hence, I can estimate the model by OLS and there is no need to resort to ML.

The results are presented in Table 5 (for the split) and Table 6 (for the spread). Tables 10, 11 and 12 shows the summary statistics for the estimated sample.^10^ Columns 1–4 in Table 5 depict the results for the OLS estimation, and columns 5–6 use a probit estimation,^11^ as the zeros in the dependent variable may be a problem to the estimation. The latter method is also a robust approach in the presence of heteroscedasticity.

**Table 5:** Spatial dependence of a split in the withholding tax rates on dividends

Variables	(1) OLS	(2) OLS	(3) OLS	(4) OLS	(5) Probit	(6) Probit
	Split in WTRs on partner’s dividends	Split in WTRs on partner’s dividends	Split in WTRs on partner’s dividends	Split in WTRs on partner’s dividends	Split in WTRs on partner’s dividends	Split in WTRs on partner’s dividends
target_lag	0.1658***	0.165***	0.166***	0.164***	0.5973***	0.5963***
	(0.0377)	(0.0382)	(0.0377)	(0.0381)	(0.1384)	(0.1383)
tieas_o	0.000669**	0.000765*	0.000962***	0.00097**	0.0036***	0.0039***
	(0.000294)	(0.000445)	(0.000319)	(0.00045)	(0.0012)	(0.0012)
tieas_d	0.0184	0.000496	0.000705**	0.000669	0.0025**	0.0029**
	(0.0283)	(0.000409)	(0.000297)	(0.00042)	(0.0011)	(0.0011)
gdp_o	1.37e−14**	1.16e−14			5.46e−14**	
	(5.85e−15)	(7.06e−15)			(2.37e−14)	
gdp_d	1.20e−14**	1.04e−14			3.51e−14*	
	(5.74e−15)	(7.01e−15)			(2.00e−14)	
gdpcap_o	6.65e−07	3.60e−07			2.44e−06	
	(4.06e−07)	(5.26e−07)			(1.61e−06)	
gdpcap_d	6.68e−07*	3.11e−07			3.26e−06**	
	(3.92e−07)	(5.08e−07)			(1.64e−06)	
LNgdp_o			− 0.116**	− 0.116*		− 0.3857**
			(0.0459)	(0.0673)		(0.1620)
LNgdp_d			− 0.0807*	− 0.119*		− 0.2545
			(0.0477)	(0.0694)		(0.1678)
LNgdpcap_o			0.134***	0.132**		0.4598***
			(0.0479)	(0.0652)		(0.1686)
LNgdpcap_d			0.0971*	0.118*		0.3151*
			(0.0497)	(0.0672)		(0.1735)
OECD_o	− 0.0331	− 0.0358	− 0.0363	− 0.0391	− 0.1393	− 0.1473*
	(0.0236)	(0.0244)	(0.0236)	(0.0244)	(0.0850)	(0.0849)
OECD_d	− 0.0286	− 0.0318	− 0.0317	− 0.0349	− 0.1211	− 0.1248
	(0.0243)	(0.0252)	(0.0243)	(0.0252)	(0.0870)	(0.0869)
OECD_pair	− 0.0159	− 0.0154	− 0.0160	− 0.0153	− 0.0607	− 0.0617
	(0.0341)	(0.0342)	(0.0340)	(0.0342)	(0.1284)	(0.1283)
EU_o	− 0.0157	− 0.0112	− 0.0346	− 0.0191	− 0.0419	− 0.1093
	(0.0247)	(0.0256)	(0.0249)	(0.0257)	(0.0827)	(0.0834)
EU_d	− 0.0143	− 0.00581	− 0.0301	− 0.0122	− 0.0402	− 0.0954
	(0.0242)	(0.0252)	(0.0244)	(0.0253)	(0.0785)	(0.0789)
EU_pair	− 0.0177	− 0.0177	− 0.0175	− 0.0177	− 0.1005	− 0.0990
	(0.0371)	(0.0401)	(0.0371)	(0.0373)	(0.1388)	(0.1388)
taxhaven_o	–	–	–	–	− 0.6210	− 0.6265
	–	–	–	–	(0.4913)	(0.4917)
taxhaven_d	–	–	–	–	− 0.4561	− 0.4639
	–	–	–	–	(0.4764)	(0.4777)
taxhaven_pair	0.00094***	− 0.0603	− 0.0592	− 0.0599	− 0.3217	− 0.3209
	(0.000314)	(0.0512)	(0.0511)	(0.0512)	(0.2280)	(0.2280)
taxdividends_o	0.0213	− 0.000879	0.0125	− 0.00583	0.0205	0.0017
	(0.0287)	(0.0318)	(0.0280)	(0.0320)	(0.1085)	(0.1074)
taxdividends_d	0.00094***	0.0177	0.0170	0.00960	0.0164	− 0.0003
	(0.000314)	(0.0320)	(0.0284)	(0.0322)	(0.1165)	(0.1153)
Time-fixed effects	Yes	Yes	Yes	Yes	Yes	Yes
Home-fixed effects	Yes	Yes	Yes	Yes	Yes	Yes
Host-fixed effects	Yes	Yes	Yes	Yes	Yes	Yes
Source-region-year fixed effects	No	Yes	No	Yes	No	No
Target-region-year fixed effects	No	Yes	No	Yes	No	No
Observations	34,396	34,396	34,396	34,396	33,676	33,676
R-squared	0.359	0.359	0.359	0.359		
Pseudo-R-squared					0.2985	0.2985

Robust standard errors in parentheses are clustered by country-pair ***p < 0.01; **p < 0.05; *p < 0.1

The dependent variable is a dummy variable having the value of unity if a country has asymmetric (multi-rated) taxes on dividends in the double tax treaty with its partner, i.e., it prescribes different rates on the withholding tax rates on portfolio and participation dividends

**Table 6:** Spatial dependence of a spread between the withholding tax rates on portfolio and participation dividends

Variables	(1)	(2)	(3)	(4)
	Spread of WTR on partner’s dividends	Spread of WTR on partner’s dividends	Spread of WTR on partner’s dividends	Spread of WTR on partner’s dividends
target_lag	0.217***	0.215***	0.217***	0.215***
	(0.0260)	(0.0262)	(0.0260)	(0.0262)
tieas_o	8.52e−05**	6.97e−05	8.15e−05**	7.93e−05
	(3.46e−05)	(4.85e−05)	(3.54e−05)	(4.99e−05)
tieas_d	0.000101***	7.55e−05	9.48e−05**	9.50e−05*
	(3.83e−05)	(5.18e−05)	(3.91e−05)	(5.27e−05)
gdp_o	6.31e−16*	4.72e−16		
	(3.65e−16)	(4.74e−16)		
gdp_d	8.12e−16**	1.20e−15**		
	(3.91e−16)	(5.82e−16)		
gdpcap_o	1.33e−07***	7.62e−08		
	(4.44e−08)	(5.95e−08)		
gdpcap_d	1.18e−07***	1.13e−07**		
	(4.44e−08)	(5.68e−08)		
LNgdp_o			− 0.00282	− 0.00488
			(0.00504)	(0.00715)
LNgdp_d			− 0.00843**	− 0.00738
			(0.00417)	(0.00603)
LNgdpcap_o			0.00189	0.00292
			(0.00521)	(0.00695)
LNgdpcap_d			0.00799*	0.00825
			(0.00425)	(0.00579)
OECD_o	− 0.00588**	− 0.00597**	− 0.00619**	− 0.00591**
	(0.00257)	(0.00262)	(0.00258)	(0.00263)
OECD_d	− 0.00577**	− 0.00608**	− 0.00602**	− 0.00609**
	(0.00246)	(0.00252)	(0.00247)	(0.00252)
OECD_pair	0.00221	0.00223	0.00219	0.00223
	(0.00335)	(0.00337)	(0.00335)	(0.00337)
EU_o	− 0.00284	− 0.00287	− 0.00347	− 0.00303
	(0.00237)	(0.00245)	(0.00237)	(0.00244)
EU_d	− 0.00313	− 0.00289	− 0.00428**	− 0.00350
	(0.00214)	(0.00222)	(0.00214)	(0.00221)
EU_pair	0.00314	0.00318	0.00317	0.00318
	(0.00372)	(0.00374)	(0.00372)	(0.00374)
taxhaven_o	–	–	–	–
	–	–	–	–
taxhaven_d	–	–	–	–
	–	–	–	–
taxhaven_pair	− 0.00406	− 0.00414	− 0.00407	− 0.00412
	(0.00561)	(0.00562)	(0.00561)	(0.00562)
taxdividends_o	0.00607**	0.00423	0.00469	0.00293
	(0.00309)	(0.00341)	(0.00308)	(0.00344)
taxdividends_d	0.00396	0.00149	0.00247	0.000195
	(0.00348)	(0.00374)	(0.00344)	(0.00376)
Time-fixed effects	Yes	Yes	Yes	Yes
Home-fixed effects	Yes	Yes	Yes	Yes
Host-fixed effects	Yes	Yes	Yes	Yes
Source-region-year fixed effects	No	Yes	No	Yes
Target-region-year fixed effects	No	Yes	No	Yes
Observations	29,876	29,875	29,876	29,875
R-squared	0.428	0.428	0.428	0.428

Robust standard errors in parentheses are clustered by country-pair ***p < 0.01; **p < 0.05; *p < 0.1

The dependent variable is the spread between the tax treaty withholding rate on portfolio and participation dividends in the host countries

From Table 5, one can see that the target lag term is positive and statistically significant across all specifications, indicating that countries look at whether their peers have multi-rated treaty withholding taxes on dividends, and if yes, they are more likely to have such splits as well. In other words, the multi-rated treaties may be influenced by the existing asymmetry in the double tax treaties by any of the two signatory countries with the peers of the other one. Therefore, there may be a spatial dependence on the rates of the countries’ peers than can be a driving factor for setting multi-rates.

Moreover, it can be seen that the findings from Sect. 3 can be validated even after taking into account the spatial dependence. In other words, countries are more likely to negotiate a high spread between the withholding tax rates in the portfolio and participation dividends in their double tax treaties if they are worried about tax avoidance and tax evasion.

Interestingly, OECD countries do not have a higher probability of having a split. This is surprising to the extent that one may expect that OECD countries, which may prefer the OECD Model Tax Convention^12^ as their guideline when concluding double tax treaties, decide on having different WTRs on portfolio and participation dividends. In contrast to the UN Model Convention^13^ that leaves the percentages open to be established during the bilateral negotiations, there is a significant difference in the OECD Model Convention, namely the differentiation in the withholding tax rates between the dividend types. The OECD Model Convention suggests different withholding tax rates for portfolio dividends (15%) and for participation dividends (5%). However, the reason for the absence of such an effect on the decision to have split rates may be connected with the fact that this effect is captured by the positive coefficient of the target lag. Apparently, when deciding to have different WTRs on dividends, countries look rather at their peers than at the fact whether their treaty partner is an OECD member or not.

Table 6 examines the spatial dependence of the spread in the withholding tax rates on portfolio and participation dividends. It reproduces Table 4,^14^ but includes the target lag. The coefficients remain unchanged, and one can still see that if countries are worried about tax avoidance and tax evasion (i.e., they have a high number of TIEAs), they are more likely to negotiate a high spread between the withholding tax rates in the portfolio and participation dividends in their double tax treaties. What is more, in line with the results in Table 4, OECD countries have a lower spread between the WTRs on dividends. When it comes to the target lag, the coefficient is positive and statistically significant across all columns, indicating that countries are more likely to have a high spread between the WTRs on portfolio and participation dividends if their peers have a high spread as well.

Overall, both Tables 5 and 6 show that countries are influenced by the already signed tax treaties and the withholding tax rates negotiated by their peers, when deciding on having split withholding tax rates in their double tax treaties, while the spread itself (i.e., the difference between the portfolio and participation dividends negotiated in the tax treaty) is also affected by the peer countries.

## Conclusion

Out of all double tax treaties (DTTs) in force in 2012, around 41% are symmetric (single-rated) and 59% are asymmetric (multi-rated), i.e., they prescribe different dividend withholding tax rates (WTRs) depending on the foreign investor’s ownership fraction. To my knowledge, this is the first, and so far only, paper dealing with this phenomenon, namely why some countries in their DTTs prefer homogenous withholding tax rates over separate rates for participation and portfolio dividends.

The paper also fills a gap in the literature by asking why and to what extent tax rates of cross-border flows such as dividends have survived over the years. With some qualifications applying, the international tax competition models would predict zero, or at least declining rates. Interestingly, the trends differ for the WTR on participation and portfolio dividends. While in the case of participation dividends a downward trend after 1990 may be observed, portfolio dividends are characterized by an upward trend in the years before mid-1990 and remain relatively stable after that. Since the early 1980s, tax treaty WTRs on portfolio dividends have on average increased by about 5.5%, while the average rate on participating dividends has fallen almost by 19.5% until 2012.

Further, the paper provides a possible explanation for the higher WTR on portfolio dividends and it may be connected to the increased concern about tax avoidance and tax evasion. In a theoretical model, I demonstrate why home countries may have an interest in a high withholding tax rate in the host country, even though they do not receive the revenue from this tax. In particular, this is the case for the WTR on portfolio dividends. The high WTR increases investor’s honesty and thus his declared income and the tax revenue in the home country. The empirical section confirms that countries that are more worried about tax avoidance and tax evasion are more likely to negotiate a higher treaty WTR on portfolio dividends in the host country. As treaty shopping is more difficult for portfolio dividends and the high WTRs cannot be avoided so easily, there is no incentive for the countries as in the case of participation dividends to set their treaty tax rates lower. Also, the increasing spread between the WTR of the portfolio and participation dividends may be an indicator that some countries are increasingly worried about tax avoidance and tax evasion in the case of portfolio dividends but are willing to reduce rates for participation dividends where tax avoidance is a much lesser issue.

Moreover, the paper does not only look into possible determinants of the withholding tax rates on dividends. It also tries to answer the question why there are different rates for participation and portfolio dividends in many double tax treaties. One explanation for the decision to have different WTRs on dividends may be connected with the fact that countries look at whether their peers have asymmetric rates on the treaty withholding tax rates on dividends, and if yes, they are more likely to have such splits as well. There is also confirming evidence that countries are influenced by the already signed tax treaties and the withholding tax rates negotiated by their peers, when deciding on having split withholding tax rates in their double tax treaties, while the spread itself (i.e., the difference between the WTRs on portfolio and participation dividends negotiated in the tax treaty) is also affected by the peer countries.

The paper deals with an understudied area and identifies one phenomenon—why some countries in their double tax treaties prefer homogenous withholding tax rates over separate rates for participation and portfolio dividends. Therefore, it offers many opportunities for future research on the topic. For instance, one can try to find any further reasons for the differentiation in the WTRs and the different development of the WTRs on dividends over time. Moreover, in order to completely eliminate the concerns of possible endogeneity issues, one can also use other proxies for the extent to which countries are worried about tax avoidance and tax evasion. It may also be interesting to conduct the analysis on a more disaggregated level and see what motivates the decision to have split rates on a country level. In case data with dividend payments are available at the necessary country level and for a long period of time, one can also measure the effect of the WTRs on dividends on the disposition of foreign subsidiary operating income or the composition of dividend payments. What is more, with accessible disaggregated data another possible extension may be to look at the composition of the countries’ tax revenue and the part coming from the two types of the WTRs on dividends. Finally, the analysis could be extended to the other two types of WTRs, namely the interest and royalty payment, in order to investigate the possible implications on them.

## Figures and Tables

**Table 7:**
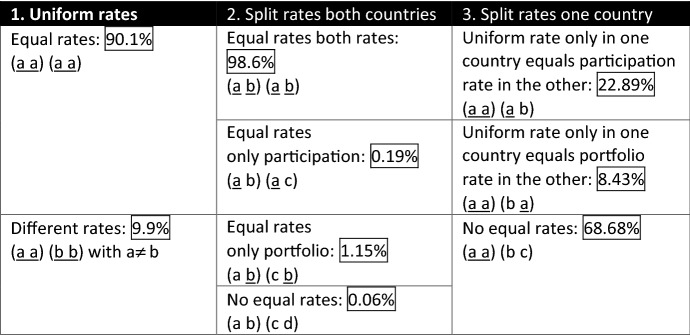
Constellations for the withholding tax rates on dividends in the double tax treaties in 2005

**Table 8:**
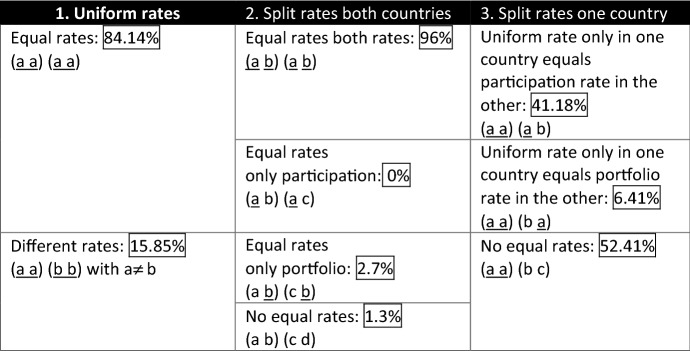
Constellations for the withholding tax rates on dividends in the double tax treaties in 1980

## Appendix 1: Theoretical model

“Appendix 1” derives a theoretical model, which uses the feature that portfolio investors are usually taxed using the credit system, and shows why the home country may have an interest in a high withholding tax rate on dividends in the host country.

Consider an investor from home country A receiving dividends from host country B. His net return function, $\pi$, is described as

1 $$\pi = \alpha D\left( {1 - \tau } \right) + T_{\text{C}} - \alpha tD + \left( {1 - \alpha } \right)D\left( {1 - \tau } \right) - D\phi \left( {1 - \alpha } \right)$$

where $\alpha$ denotes the share of dividends that is declared in the home country (i.e., an investor’s honesty factor); $D$ are the received dividends^15^;$\tau$ is the withholding tax rate on dividends in the host country B; $T_{\text{C}}$ is the tax credit in home country A with $T_{\text{C}} = \hbox{min} \left( {t\alpha D;\tau \alpha D} \right)$; *t* depicts the income tax rate in the home country A; $D\phi \left( {1 - \alpha } \right)$ captures the cost of lying about the share of declared income; $\phi$ is assumed to be a weakly increasing, convex function of non-declared dividends $\phi = D\phi \left( {1 - \alpha } \right)$.

By assuming that $\tau < t$, it follows that $T_{\text{C}} = \tau \alpha D$. Taking this into account and taking the first-order condition with respect to investor’s honesty, $\alpha$, yields:

2 $$t - \tau = \phi^{{\prime }} ,$$

The interpretation of this expression is straightforward. It shows that the marginal benefit of the investor from paying the low withholding tax rather than the higher home tax has to equal his marginal cost of non-reporting dividends.

Now, let us turn to the effect of the withholding tax rate in the host country, $\tau$, on the investor’s honesty, $\alpha$. Forming the first-order condition for maximization of $\pi$ w.r.t. $\alpha$^16^and deriving the comparative static yields:

3 $$\frac{{{\text{d}}\alpha }}{{{\text{d}}\tau }} = \frac{1}{{\phi^{{{\prime \prime }}} }},$$

which is positive, as $\phi^{{{\prime \prime }}} > 0$ due to convexity. The fact that honesty increases in $\tau$ indicates why the home country may have an interest in a high withholding tax rate on dividends in the host country. When $\tau$ goes up, investor’s honesty increases as well and thereby also the declared income at home. Therefore, this may lead to more tax revenue in the home country. The above-mentioned expression illustrates why withholding tax rates on dividends may be introduced in double tax treaties. At the same time, a higher $\tau$ may be as it triggers a high tax credit.

To evaluate the combined effect, consider the home tax revenue function:

4 $$T = \alpha Dt - T_{\text{C}} .$$

Note that here an implicit assumption is that $\phi$ is not part of the revenue (which could be the case if some of $\phi$ represents fines). Noting that $T_{\text{C}} = \tau \alpha D$ and taking the first-order condition of T w.r.t. $\tau$ yields:

5 $$\frac{{{\text{d}}T}}{{{\text{d}}\tau }} = \left[ {\frac{{{\text{d}}\alpha }}{{{\text{d}}\tau }}\left( {t - \tau } \right) - \alpha } \right]D.$$

From (3), we know that $\frac{{{\text{d}}\alpha }}{{{\text{d}}\tau }} = \frac{1}{{\phi^{{{\prime \prime }}} }}$. Therefore,

6 $$\frac{{{\text{d}}T}}{{{\text{d}}\tau }} = \left[ {\frac{1}{{\phi^{{{\prime \prime }}} }}\left( {t - \tau } \right) - \alpha } \right]D.$$

The derived expression includes two effects. The second term of (6), $\alpha D,$ is the direct effect: A higher $\tau$ decreases tax revenue, as the tax credit on honest dividends increases. The first term of (6), $\frac{1}{{\phi^{{{\prime \prime }}} }}\left( {t - \tau } \right)D,$ is the positive effect of additional honesty. With a larger $\frac{{{\text{d}}T}}{{{\text{d}}\tau }}$, the home country has a higher willingness to accept a high WTR by the source country. The total effect of the WTR on the tax revenue in the home country is positive if the effect coming from the additional honesty is bigger than the tax credit that has to be paid as a result.

For a convex function of $\phi$ in the form of $\phi = D\theta \left[ {\left( {1 - \alpha } \right)} \right]^{x}$ with *x* > 1, the first derivative will be $\phi^{{\prime }} = D\theta x\left[ {\left( {1 - \alpha } \right)} \right]^{x - 1}$ and the second derivative accordingly $\phi^{{{\prime \prime }}} = D\theta \left( {x - 1} \right)x\left[ {\left( {1 - \alpha } \right)} \right]^{x - 2}$.

Since home country wants to maximize its tax revenue *T*, its preferred $\tau$ for a given t is found via setting $\frac{{{\text{d}}T}}{{{\text{d}}\tau }} = 0$. From (6), it follows that $\phi^{{{\prime \prime }}} = \frac{t - \tau }{\alpha }$.

Hence,

7 $$t - \tau = \alpha Dx\left( {x - 1} \right)\theta (1 - \alpha )^{x - 2} .$$

Therefore, if $\theta$ or $x$ go up, the preferred $\tau$ of the home country goes down. In other words, if lying gets costlier (for example, via exchange of information), the withholding tax rate of the host country is less relevant for the home country, as the honesty of the investor has increased. Overall, one can see that there is a negative connection between the tax avoidance in the home country and the withholding tax rate of the partner country.

## Appendix 2: Constellations for the withholding tax rates on dividends in the double tax treaties

This appendix reproduces Table 1 by looking at the development of the withholding tax rates on portfolio and participation dividends over time and depicting the same constellations for 2005 and 1980 (Tables 7, 8).

## Appendix 3: Summary statistics

This appendix depicts the summary statistics for the estimated samples in Tables 2, 3, 4, 5, 6, 9, 10, 11 and 12.

**Table 9:** Summary statistics for Tables 2, 3 and 4

Variable	Obs.	Mean	SD	Min	Max
WHT on host’ portfolio dividends	33,310	0.1240471	0.0515946	0	0.47
WHT on host’ participation dividends	33,310	0.0759778	0.0524172	0	0.47
Spread between the WTRs	33,310	0.0480694	0.0479371	0	0.2
gdp_o	33,310	8.88e + 11	1.96e + 12	8.47e + 08	1.62e + 13
gdp_d	33,310	8.76e + 11	1.94e + 12	8.47e + 08	1.62e + 13
gdpcap_o	33,310	24958.19	23308.1	162.8132	113738.7
gdpcap_d	33,310	24834.37	23201.15	162.8132	113738.7
OECD_o	33,310	0.4775743	0.4983006	0	1
OECD_d	33,310	0.4587211	0.4995043	0	1
OECD_pair	33,310	0.1959472	0.396934	0	1
EU_o	33,310	0.3666467	0.4818961	0	1
EU_d	33,310	0.3611828	0.4803506	0	1
EU_pair	33,310	0.1254278	0.3312083	0	1
taxhaven_o	33,310	0.1124887	0.3159716	0	1
taxhaven_d	33,310	0.1217352	0.3269847	0	1
taxhaven_pair	33,310	0.0167217	0.1282286	0	1
taxdividends_o	33,310	0.1411164	0.1091825	0	0.55
taxdividends_d	33,310	0.139616	0.109793	0	0.55
tieas_o	33,310	2.761693	6.920261	0	37
tieas_d	33,310	2.707926	6.856868	0	37

**Table 10:** Summary statistics for Table 5 (1)

Variable	Obs.	Mean	Std. Dev.	Min	Max
split	101,303	0.4744381	0.4993486	0	1
target_lag	101,303	0.2404886	0.271204	0	1
gdp_o	101,303	8.04e + 11	1.50e + 12	1.26e + 07	1.62e + 13
gdp_d	101,303	6.04e + 11	1.50e + 12	1.26e + 07	1.62e + 13
gdpcap_o	101,303	16168.46	17854.31	47.46514	113738.7
gdpcap_d	101,303	16168.5	17854.29	40.46514	113738.7
OECD_o	101,303	0.5384836	0.4985193	0	1
OECD_d	101,303	0.5253349	0.4993602	0	1
OECD_pair	101,303	0.2568729	0.4369109	0	1
EU_o	101,303	0.3119947	0.46331	0	1
EU_d	101,303	0.3119947	0.46331	0	1
EU_pair	101,303	0.0865917	0.2812372	0	1
taxhaven_o	101,303	0.1096216	0.3124191	0	1
taxhaven_d	101,303	0.1096315	0.3124314	0	1
taxhaven_pair	101,303	0.0125564	0.1113501	0	1
tieas_o	101,303	0.9322922	4.199315	0	37
tieas_d	101,303	0.9322922	4. 199315	0	37

**Table 11:** Summary statistics for Table 5 (1)–(4)

Variable	Obs.	Mean	Std. Dev.	Min	Max
split	34,396	0.5597744	0.4964214	0	1
target_lag	34,396	0.3104911	0.2947316	0	1
gdp_o	34,396	8.79e + 11	1.96e + 12	8.47e + 08	1.62e + 13
gdp_d	34,396	8.79e + 11	1.96e + 12	8.47e + 08	1.62e + 13
gdpcap_o	34,396	24689.39	23141.99	162.8132	113738.7
gdpcap_d	34,396	24689.39	23140.99	162.8132	113738.7
OECD_o	34,396	0.4692215	0.4990584	0	1
OECD_d	34,396	0.4569136	0.4981473	0	1
OECD_pair	34,396	0.1926968	0.3944227	0	1
EU_o	34,396	0.3609141	0.4802726	0	1
EU_d	34,396	0.3609141	0.44802726	0	1
EU_pair	34,396	0.1243168	0.3299474	0	1
taxhaven_o	34,396	0.120421	0.3254579	0	1
taxhaven_d	34,396	0.120421	0. 3254579	0	1
taxhaven_pair	34,396	0.0178509	0.1324114	0	1
taxdividends_o	34,396	0.1390637	0.1099107	0	0.55
taxdividends_d	34,396	0.1390637	0. 1099107	0	0.55
tieas_o	34,396	2.698366	6.843898	0	37
tieas_d	34,396	2.698366	6.843898	0	37

**Table 12:** Summary statistics for Table 5 (5)–(6)

Variable	Obs.	Mean	Std. Dev.	Min	Max
split	33,676	0.5663974	0.4955791	0	1
target_lag	33,676	0.3155696	0.2947642	0	1
gdp_o	33,676	8.85e + 11	1.98e + 12	8.47e + 08	1.62e + 13
gdp_d	33,676	8.85e + 11	1.98e + 12	8.47e + 08	1.62e + 13
gdpcap_o	33,676	24835.53	23121.76	162.8132	113738.7
gdpcap_d	33,676	24835.53	23121.76	162.8132	113738.7
OECD_o	33,676	0.4723245	0.4992409	0	1
OECD_d	33,676	0.4597636	0.4983858	0	1
OECD_pair	33,676	0.1968167	0.3975985	0	1
EU_o	33,676	0.3633745	0.4809785	0	1
EU_d	33,676	0.3633745	0.4809785	0	1
EU_pair	33,676	0.1269747	0.3329496	0	1
taxhaven_o	33,676	0.1215703	0.3267937	0	1
taxhaven_d	33,676	0.1215703	0.3267937	0	1
taxhaven_pair	33,676	0.017995	0.132935	0	1
taxdividends_o	33,676	0.1387755	0.1099384	0	0.55
taxdividends_d	33,676	0.1387755	0.1099384	0	0.55
tieas_o	33,676	2.707685	6.860425	0	37
tieas_d	33,676	2.707685	6.860425	0	37

## Appendix 4: Robustness checks

The first part of the appendix reproduces Table 2, but uses another proxy for the extent to which countries are worried about tax avoidance and tax evasion is the tax morale variable from the World Values Survey (WVS 2015) that asks the question: “Do you justify cheating on taxes if you have the chance?” and takes the values between 1 (always) and 10 (never). The hypothesis is that countries, whose population is more likely to cheat on their taxes, may be worried about tax avoidance and tax evasion and therefore have an interest in a high tax treaty withholding rate on portfolio dividends in the host country. The results in Table 13 remain unchanged, and one can confirm the hypothesis that countries with population having a low tax morale are expected to have an interest in a high treaty withholding tax rate on portfolio dividends in the host country.

**Table 13:** Tax treaty withholding rates on portfolio dividends in the host country with a different proxy

Variables	(1) WTR on host’ portfolio dividends	(2) WTR on host’ portfolio dividends
tax_morale_o	− 2.438***	− 1.663***
	(0.310)	(0.551)
tax_morale_d	− 0.778	− 1.376*
	(0.684)	(0.778)
gdp_o	− 1.64e−16	
	(2.36e−16)	
gdp_d	3.53e−16	
	(2.89e−16)	
gdpcap_o	1.37e−07***	
	(4.70e−08)	
gdpcap_d	7.60e−08	
	(4.72e−08)	
ln_gdp_o		0.0137*
		(0.00744)
ln_gdp_d		− 0.00875
		(0.00577)
ln_gdpcap_o		− 0.0132*
		(0.00779)
ln_gdpcap_d		0.0100*
		(0.00604)
OECD_o	− 0.00475	− 0.00463
	(0.00289)	(0.00289)
OECD_d	− 0.00306	− 0.00311
	(0.00288)	(0.00288)
OECD_pair	− 0.00281	− 0.00281
	(0.00322)	(0.00322)
EU_o	− 0.00505**	− 0.00451**
	(0.00212)	(0.00214)
EU_d	− 0.00500**	− 0.00611***
	(0.00210)	(0.00209)
EU_pair	0.00621*	0.00622*
	(0.00355)	(0.00355)
taxhaven_o	− 0.795***	− 0.546***
	(0.0978)	(0.176)
taxhaven_d	− 0.265	− 0.458*
	(0.218)	(0.249)
taxhaven_pair	0.0127	0.0127
	(0.00831)	(0.00832)
taxdividends_o	− 0.000739	− 0.00139
	(0.00307)	(0.00305)
taxdividends_d	0.000449	4.74e−05
	(0.00360)	(0.00357)
Time-fixed effects	Yes	Yes
Home-fixed effects	Yes	Yes
Host-fixed effects	Yes	Yes
Observations	20,773	20,773
R-squared	0.468	0.468

Robust standard errors in parentheses are clustered by country-pair ***p < 0.01;**p < 0.05; *p < 0.1

The dependent variable is the tax treaty withholding rate on portfolio dividends in the host countries

The second part of the appendix reproduces Table 4. However, the dependent variable in Table 14 now is an indicator variable (multi-rated dummy), which takes the value of 1 if the treaty between home and host countries is multi-rated. The coefficients of the TIEAs remain statistically significant and positive. Further, the control variables are added to the estimation model—the withholding tax rate on portfolio dividends (in Table 15) and the withholding tax rate on participation dividends (in Table 16). One can see that the spread between the WTRs on portfolio and participation dividends increases with the WTR on portfolio dividends and decreases with the WTR on participation dividends.

**Table 14:** Split in the WTRs on dividends in the host country—robustness test with multi-rated treaty dummy as a dependent variable

Variables	(1) Multi-rated treaty	(2) Multi-rated treaty	(3) Multi-rated treaty	(4) Multi-rated treaty
tieas_o		0.000821***		0.000853***
		(0.000303)		(0.000310)
tieas_d		0.000969***		0.00101***
		(0.000324)		(0.000332)
gdp_o	1.06e−14*	1.16e−14**		
	(5.68e−15)	(5.71e−15)		
gdp_d	1.27e−14**	1.38e−14**		
	(5.76e−15)	(5.79e−15)		
gdpcap_o	8.89e−07**	7.79e−07*		
	(4.01e−07)	(4.00e−07)		
gdpcap_d	9.36e−07**	7.99e−07*		
	(4.10e−07)	(4.10e−07)		
ln_gdp_o			− 0.0982**	− 0.0891*
			(0.0484)	(0.0481)
ln_gdp_d			− 0.133***	− 0.123***
			(0.0472)	(0.0470)
ln_gdpcap_o			0.106**	0.105**
			(0.0504)	(0.0502)
ln_gdpcap_d			0.143***	0.142***
			(0.0491)	(0.0489)
OECD_o	− 0.0332	− 0.0300	− 0.0370	− 0.0331
	(0.0244)	(0.0244)	(0.0244)	(0.0244)
OECD_d	− 0.0369	− 0.0328	− 0.0408*	− 0.0361
	(0.0236)	(0.0236)	(0.0236)	(0.0236)
OECD_pair	− 0.00895	− 0.00934	− 0.00901	− 0.00939
	(0.0341)	(0.0341)	(0.0341)	(0.0341)
EU_o	− 0.0285	− 0.0251	− 0.0442*	− 0.0416*
	(0.0243)	(0.0243)	(0.0246)	(0.0246)
EU_d	− 0.0293	− 0.0255	− 0.0485**	− 0.0456*
	(0.0242)	(0.0241)	(0.0245)	(0.0244)
EU_pair	0.00660	0.00691	0.00681	0.00702
	(0.0370)	(0.0370)	(0.0370)	(0.0370)
taxhaven_o	− 0.202	− 0.204	− 0.190	− 0.202
	(0.166)	(0.166)	(0.166)	(0.166)
taxhaven_d	− 0.240	− 0.241	− 0.225	− 0.239
	(0.162)	(0.162)	(0.162)	(0.162)
taxhaven_pair	− 0.0613	− 0.0613	− 0.0611	− 0.0610
	(0.0513)	(0.0513)	(0.0513)	(0.0513)
taxdividends_o	0.0205	0.0304	0.0121	0.0250
	(0.0281)	(0.0289)	(0.0277)	(0.0286)
taxdividends_d	0.0150	0.0261	0.00483	0.0198
	(0.0274)	(0.0284)	(0.0270)	(0.0281)
Time-fixed effects	Yes	Yes	Yes	Yes
Home-fixed effects	Yes	Yes	Yes	Yes
Host-fixed effects	Yes	Yes	Yes	Yes
Observations	34,412	34,412	34,412	34,412
R-squared	0.355	0.355	0.355	0.355

Robust standard errors in parentheses are clustered by country-pair ***p < 0.01; **p < 0.05; *p < 0.1

The dependent variable is an indicator variable taking the value of 1 if the tax treaty is multi-rated

**Table 15:** Spread between the WTRs on portfolio and participation dividends in the host country—robustness test with portfolio dividends as a control variable

Variables	(1) Spread on partner’s dividends	(2) Spread on partner’s dividends	(3) Spread on partner’s dividends	(4) Spread on partner’s dividends
tieas_o		0.0000879***		0.0000806***
		(0.0000296)		(0.0000316)
tieas_d		0.0000904**		0.0000853**
		(0.0000323)		(0.0000339)
gdp_o	5.98e−16*	7.07e−16**		
	(3.17e−16)	(3.17e−16)		
gdp_d	4.73e−16	5.82e−16*		
	(3.15e−16)	(3.14e−16)		
gdpcap_o	1.43e−07***	1.30e−07***		
	(3.73e−08)	(3.62e−08)		
gdpcap_d	1.57e−07***	1.45e−07***		
	(3.98e−08)	(3.92e−08)		
ln_gdp_o			− 0.0080868***	− 0.0072253**
			(0.0029586)	(0.0029606)
ln_gdp_d			− 0.0082422***	− 0.0073349**
			(0.002963)	(0.0029542)
ln_gdpcap_o			0.064457**	0.0063926**
			(0.0030427)	(0.0030303)
ln_gdpcap_d			0.0065627**	0.0065196**
			(0.0030634)	(0.0030499)
OECD_o	− 0.004092*	− 0.00373**	− 0.004433**	− 0.0040521*
	(0.0021639)	(0.0021609)	(0.0021728)	(0.0021765)
OECD_d	− 0.0056287***	− 0.0052649***	− 0.0059216***	− 0.0055286***
	(0.0019801)	(0.0019844)	(0.0019879)	(0.0019944)
OECD_pair	0.0046236	0.0045861	0.0046059	0.0045721*
	(0.0029706)	(0.0029716)	(0.0029705)	(0.0029714)
EU_o	− 0.0024588	− 0.0020815	− 0.0033622*	− 0.0031052
	(0.0019263)	(0.0019253)	(0.001933)	(0.0019287)
EU_d	− 0.0024482	− 0.0020874	− 0.0033655*	− 0.0031216
	(0.001916)	(0.0019137)	(0.0019261)	(0.0019209)
EU_pair	0.0032835	0.0033173	0.0033352	0.0033562
	(0.0033505)	(0.0033507)	(0.0033503)	(0.0033505)
taxhaven_o	− 0.017301	− 0.0174465	− 0.0135961	− 0.0147303
	(0.0137936)	(0.0137995)	(0.0139412)	(0.0139422)
taxhaven_d	− 0.0186708	− 0.0188389	− 0.0148022	− 0.0160191
	(0.0141461)	(0.0141483)	(0.0142872)	(0.0142794)
taxhaven_pair	− 0.0021259	− 0.0021286	− 0.0021176	− 0.0021154
	(0.0039782)	(0.003982)	(0.0039822)	(0.0039856)
taxdividends_o	0.007395***	0.0085098***	0.0057079**	0.0069696**
	(0.0027357)	(0.0028209)	(0.0027099)	(0.0027685)
taxdividends_d	0.0040217*	0.0050994**	0.0022131	0.0034855
	(0.0021826)	(0.0022961)	(0.0021637)	(0.0022756)
dividends_portfolio_d	0.4457631***	0.4455629***	0.4458827***	0.4456816***
	(0.0225646)	(0.0225597)	(0.0225675)	(0.0225643)
Time-fixed effects	Yes	Yes	Yes	Yes
Home-fixed effects	Yes	Yes	Yes	Yes
Host-fixed effects	Yes	Yes	Yes	Yes
Observations	33,310	33,310	33,310	33,310
R-squared	0.535	0.535	0.535	0.535

Robust standard errors in parentheses are clustered by country-pair ***p < 0.01; **p < 0.05; *p < 0.1

The dependent variable is the spread between the tax treaty WTR on portfolio and participation dividends in the host countries

**Table 16:** Spread between the WTRs on portfolio and participation dividends in the host country—robustness test with dividends as a control variable

Variables	(1) Spread on partner’s dividends	(2) Spread on partner’s dividends	(3) Spread on partner’s dividends	(4) Spread on partner’s dividends
tieas_o		0.0001079***		0.0001153***
		(0.0000304)		(0.000031)
tieas_d		0.0001096***		0.0001163***
		(0.0000333)		(0.0000344)
gdp_o	2.55e−16	3.89e−16		
	(2.40e−16)	(2.38e−16)		
gdp_d	6.06e−16**	7.38e−16***		
	(2.75e−16)	(2.74e−16)		
gdpcap_o	1.32e−07***	1.17e−07***		
	(3.51e−08)	(3.50e−08)		
gdpcap_d	1.23e−07***	1.08e−07***		
	(3.55e−08)	(3.52e−08)		
ln_gdp_o			− 0.0055506	− 0.0043219
			(0.0042629)	(0.0042353)
ln_gdp_d			− 0.0098358**	− 0.008592**
			(0.0041717)	(0.0041509)
ln_gdpcap_o			0.0051263	0.005059
			(0.0044304)	(0.0044096)
ln_gdpcap_d			0.009817**	0.0097465**
			(0.0043349)	(0.0043159)
OECD_o	− 0.0049309**	− 0.00373**	− 0.005132**	− 0.0045835**
	(0.0020058)	(0.0020001)	(0.0020049)	(0.0019981)
OECD_d	− 0.0046832**	− 0.0044845**	− 0.0049148**	− 0.0043813**
	(0.0020119)	(0.0020125)	(0.0020112)	(0.0020101)
OECD_pair	0.0003976	0.0004416	0.0004098	0.0004554
	(0.0026659)	(0.0026671)	(0.0026658)	(0.0026667)
EU_o	− 0.0046941***	− 0.0042306**	− 0.0055633***	− 0.0051982***
	(0.0017906)	(0.0017976)	(0.001801)	(0.0018036)
EU_d	− 0.0044406**	− 0.0040014**	− 0.0057364***	− 0.0053994***
	(0.0017727)	(0.0017779)	(0.0017828)	(0.0017849)
EU_pair	0.0046335	0.0046738	0.0046622	0.0046905
	(0.0029635)	(0.0029626)	(0.0029634)	(0.0029626)
taxhaven_o	− 0.013703	− 0.0138812	− 0.0116309	− 0.013257
	(0.0098737)	(0.0098677)	(0.0099119)	(0.0099017)
taxhaven_d	− 0.0175985*	− 0.0178015*	− 0.0153587	− 0.0170126
	(0.009842)	(0.009829)	(0.0098901)	(0.0098806)
taxhaven_pair	− 0.00542	− 0.0054218	− 0.0054067	− 0.005402
	(0.0053568)	(0.0053591)	(0.0053601)	(0.0053623)
taxdividends_o	0.0019443	0.0033119	0.0007936	0.0025932
	(0.0026676)	(0.0027909)	(0.0026294)	(0.0027803)
taxdividends_d	0.0040347	0.0053436*	0.0027776	0.0045186
	(0.0027857)	(0.0029082)	(0.0027343)	(0.0028934)
dividends_participation_d	− 0.4778899***	− 0.4777832***	− 0.4780314***	− 0.477963***
	(0.0206223)	(0.0206153)	(0.0206279)	(0.0206188)
Time-fixed effects	Yes	Yes	Yes	Yes
Home-fixed effects	Yes	Yes	Yes	Yes
Host-fixed effects	Yes	Yes	Yes	Yes
Observations	33,310	33,310	33,310	33,310
R-squared	0.552	0.562	0.562	0.562

Robust standard errors in parentheses are clustered by country-pair ***p < 0.01; **p < 0.05; *p < 0.1

The dependent variable is the spread between the tax treaty WTR on portfolio and participation dividends in the host countries

The third part of the appendix reproduces Table 5. Table 17 excludes the target lag from the estimation in order to verify whether it derives the results for the coefficients on the TIEAs. Table 18 reproduces Table 5, while including only new treaties as separate observations. Despite the smaller sample, results remain unchanged.

**Table 17:** Spatial dependence of a split in the withholding tax rates on dividends

Variables	(1) OLS	(2) OLS	(3) OLS	(4) OLS	(5) Probit	(6) Probit
	Split in WTRs on partner’s dividends	Split in WTRs on partner’s dividends	Split in WTRs on partner’s dividends	Split in WTRs on partner’s dividends	Split in WTRs on partner’s dividends	Split in WTRs on partner’s dividends
target_lag						
tieas_o	0.000969***	0.000795*	0.00101***	0.000795*	0.0039***	0.0043***
	(0.000324)	(0.000451)	(0.000332)	(0.000451)	(0.0013)	(0.0013)
tieas_d	0.000821***	0.000747*	0.000853***	0.000747*	0.0031***	0.0035**
	(0.000303)	(0.000421)	(0.000310)	(0.000421)	(0.0012)	(0.0012)
gdp_o	1.38e−14	− 4.80e−15			5.57e−14**	
	(5.79e−15)	(4.32e−15)			(2.32e−14)	
gdp_d	1.16e−14	− 4.91e−15			3.46e−14*	
	(5.71e−15)	(4.28e−15)			(2.00e−14)	
gdpcap_o	7.99e−07*	− 6.23e−07			2.96e−06*	
	(4.10e−07)	(5.16e−07)			(1.63e−06)	
gdpcap_d	7.79e−07*	− 8.29e−07			3.61e−06**	
	(4.00e−07)	(5.09e−07)			(1.66e−06)	
LNgdp_o			− 0.123***	− 0.131*		− 0.4023*
			(0.0470)	(0.0688)		(0.1671)
LNgdp_d			− 0.0891*	− 0.131*		− 0.2730
			(0.0481)	(0.0703)		(0.1703)
LNgdpcap_o			0.142***	0.152**		0.4821***
			(0.0489)	(0.0666)		(0.1737)
LNgdpcap_d			0.105**	0.131*		0.3332*
			(0.0502)	(0.0681)		(0.1760)
OECD_o	− 0.0328	− 0.0416*	− 0.0361	− 0.0416*	− 0.1322	− 0.1400
	(0.0236)	(0.0245)	(0.0236)	(0.0245)	(0.0850)	(0.0850)
OECD_d	− 0.0300	− 0.0388	− 0.0331	− 0.0388	− 0.1213	− 0.1249
	(0.0244)	(0.0252)	(0.0244)	(0.0252)	(0.0870)	(0.0862)
OECD_pair	− 0.00934	− 0.00873	− 0.00939	− 0.00873	− 0.0449	− 0.0461
	(0.0341)	(0.0342)	(0.0341)	(0.0342)	(0.1284)	(0.1272)
EU_o	− 0.0255	− 0.0283	− 0.0456*	− 0.0283	− 0.0810	− 0.1521*
	(0.0241)	(0.0253)	(0.0244)	(0.0253)	(0.0798)	(0.0806)
EU_d	− 0.0251	− 0.0241	− 0.0416*	− 0.0241	− 0.0821	− 0.1375*
	(0.0243)	(0.0254)	(0.0246)	(0.0254)	(0.0764)	(0.0771)
EU_pair	0.00691	0.00667	0.00702	0.00667	− 0.0061	− 0.0046
	(0.0370)	(0.0372)	(0.0370)	(0.0372)	(0.1361)	(0.1361)
taxhaven_o	–	–	–	–	− 0.7869	− 0.7922
	–	–	–	–	(0.4991)	(0.4996)
taxhaven_d	–	–	–	–	− 0.6109	− 0.6129
	–	–	–	–	(0.4846)	(0.4863)
taxhaven_pair	− 0.0613	− 0.0617	− 0.0610	− 0.0617	− 0.3128	− 0.3119
	(0.0513)	(0.0514)	(0.0513)	(0.0514)	(0.2279)	(0.2279)
taxdividends_o	0.0261	− 0.00277	0.0198	− 0.00277	0.0567	0.0360
	(0.0284)	(0.0322)	(0.0281)	(0.0322)	(0.1089)	(0.1080)
taxdividends_d	0.0304	0.0100	0.0250	0.0100	0.0544	0.0354
	(0.0289)	(0.0325)	(0.0286)	(0.0325)	(0.1158)	(0.1147)
Time-fixed effects	Yes	Yes	Yes	Yes	Yes	Yes
Home-fixed effects	Yes	Yes	Yes	Yes	Yes	Yes
Host-fixed effects	Yes	Yes	Yes	Yes	Yes	Yes
Source-region-year fixed effects	No	Yes	No	Yes	No	No
Target-region-year fixed effects	No	Yes	No	Yes	No	No
Observations	34,412	34,412	34,412	34,412	33,692	33,692
R-squared	0.355	0.355	0.355	0.355		
Pseudo-R-squared					0.2943	0.2942

Robust standard errors in parentheses are clustered by country-pair ***p < 0.01; **p < 0.05; *p < 0.1

The dependent variable is a dummy variable having the value of unity if a country has asymmetric (multi-rated) taxes on dividends in the double tax treaty with its partner, i.e., it prescribes different rates on the withholding tax rates on portfolio and participation dividends

**Table 18:** Spatial dependence of a split in the withholding tax rates on dividends

Variables	(1) OLS	(2) OLS	(3) OLS	(4) OLS	(5) Probit	(6) Probit
	Split in WTRs on partner’s dividends	Split in WTRs on partner’s dividends	Split in WTRs on partner’s dividends	Split in WTRs on partner’s dividends	Split in WTRs on partner’s dividends	Split in WTRs on partner’s dividends
target_lag	0.156*	0.148*	0.128	0.121	1.219**	1.077**
	(0.0815)	(0.0864)	(0.0806)	(0.0871)	(0.478)	(0.484)
tieas_o	− 0.00180	− 0.00286	− 0.000943	− 0.00287	− 0.0297	− 0.00671
	(0.00304)	(0.00568)	(0.00305)	(0.00558)	(0.0224)	(0.0233)
tieas_d	− 0.00237	− 0.00129	− 0.00135	− 0.00156	− 0.0344	− 0.0106
	(0.00306)	(0.00570)	(0.00307)	(0.00554)	(0.0254)	(0.0265)
gdp_o	4.75e−14	− 4.80e−15			5.25e−13	
	(5.62e−14)	(4.32e−15)			(4.02e−13)	
gdp_d	4.88e−14	− 4.91e−15			2.05e−13	
	(5.05e−14)	(4.28e−15)			(2.48e−13)	
gdpcap_o	− 6.72e−06**	− 9.04e−06**			− 3.03e−05	
	(3.25e−06)	(4.00e−06)			(1.86e−05)	
gdpcap_d	− 2.14e−06	− 6.76e−06*			− 2.49e−06	
	(3.31e−06)	(4.10e−06)			(1.93e−05)	
LNgdp_o			− 0.242	− 0.0359		− 0.656
			(0.201)	(0.292)		(1.126)
LNgdp_d			− 0.0903	0.0276		− 0.00727
			(0.216)	(0.303)		(1.146)
LNgdpcap_o			0.362*	0.177		2.022*
			(0.203)	(0.267)		(1.070)
LNgdpcap_d			0.237	0.0922		1.397
			(0.217)	(0.279)		(1.051)
OECD_o	− 0.212*	− 0.220	− 0.236*	− 0.214	− 1.151	− 1.193*
	(0.126)	(0.171)	(0.127)	(0.168)	(0.711)	(0.702)
OECD_d	− 0.191	− 0.156	− 0.206	− 0.145	− 1.184*	− 1.181*
	(0.131)	(0.163)	(0.131)	(0.161)	(0.659)	(0.657)
OECD_pair	− 0.209***	− 0.165*	− 0.214***	− 0.164*	− 1.436**	− 1.415**
	(0.0804)	(0.0885)	(0.0808)	(0.0896)	(0.612)	(0.616)
EU_o	− 0.0385	0.194	− 0.0887	0.173	2.056**	1.570*
	(0.188)	(0.252)	(0.193)	(0.247)	(0.900)	(0.937)
EU_d	0.00816	0.245	− 0.0520	0.216	2.124**	1.706*
	(0.194)	(0.263)	(0.203)	(0.259)	(0.936)	(0.956)
EU_pair	0.0798	0.0737	0.0837	0.0920	1.264**	1.280**
	(0.0950)	(0.0998)	(0.0943)	(0.0989)	(0.635)	(0.652)
taxhaven_o	–	–	–	–	0.134	− 1.289
	–	–	–	–	(0.823)	(0.990)
taxhaven_d	–	–	–	–	0.794	− 0.615
	–	–	–	–	(1.012)	(1.141)
taxhaven_pair	− 0.133	− 0.135	− 0.140	− 0.145	− 1.347**	− 1.400**
	(0.0964)	(0.113)	(0.0961)	(0.116)	(0.543)	(0.553)
taxdividends_o	− 0.0223	− 0.590	− 0.0311	− 0.549	0.510	− 0.146
	(0.516)	(0.599)	(0.515)	(0.590)	(2.895)	(2.870)
taxdividends_d	0.00170	− 0.508	− 0.0661	− 0.508	0.776	0.477
	(0.519)	(0.611)	(0.517)	(0.600)	(2.920)	(2.898)
Time-fixed effects	Yes	Yes	Yes	Yes	Yes	Yes
Home-fixed effects	Yes	Yes	Yes	Yes	Yes	Yes
Host-fixed effects	Yes	Yes	Yes	Yes	Yes	Yes
Source-region-year fixed effects	No	Yes	No	Yes	No	No
Target-region-year fixed effects	No	Yes	No	Yes	No	No
Observations	1118	1086	1118	1086	811	811
R-squared	0.552	0.688	0.552	0.686		
Pseudo-R-squared				0.1836	0.474	0.474

Robust standard errors in parentheses are clustered by country-pair ***p < 0.01; **p < 0.05; *p < 0.1

The dependent variable is a dummy variable having the value of unity if a country has asymmetric (multi-rated) taxes on dividends in the double tax treaty with its partner, i.e., it prescribes different rates on the withholding tax rates on portfolio and participation dividends

The last part of the appendix with Table 19 reproduces Table 6, but includes only new treaties as separate observations. Despite the smaller sample, results remain unchanged.

**Table 19:** Spatial Dependence of a Spread in the Withholding Tax Rates on Dividends

Variables	(1) Spread of WTRs on partner’s dividends	(2) Spread on WTRs on partner’s dividends	(3) Spread of WTRs on partner’s dividends	(4) Spread of WTRs on partner’s dividends
target_lag	0.132**	0.0422	0.126**	0.0523
	(0.0569)	(0.0586)	(0.0570)	(0.0596)
tieas_o	− 5.76e−05	0.000173	− 7.88e−05	− 0.000178
	(0.000398)	(0.000637)	(0.000407)	(0.000627)
tieas_d	− 0.000227	− 0.000368	− 0.000237	− 0.000551
	(0.000388)	(0.000630)	(0.000395)	(0.000636)
gdp_o	− 6.43e−15	− 1.78e−14**		
	(5.26e−15)	(6.91e−15)		
gdp_d	2.56e−16	− 1.63e−14*		
	(5.40e−15)	(9.21e−15)		
gdpcap_o	− 2.97e−07	− 1.18e−06**		
	(3.39e−07)	(4.76e−07)		
gdpcap_d	1.16e−07	− 9.37e−08		
	(3.16e−07)	(4.32e−07)		
LNgdp_o			− 0.00676	− 0.0108
			(0.0227)	(0.0313)
LNgdp_d			− 0.00336	− 0.000968
			(0.0203)	(0.0293)
LNgdpcap_o			0.00196	− 0.0156
			(0.0215)	(0.0289)
LNgdpcap_d			0.0121	− 0.00811
			(0.0205)	(0.0275)
OECD_o	− 0.0224**	− 0.0130	− 0.0227**	− 0.0153
	(0.0112)	(0.0157)	(0.0113)	(0.0153)
OECD_d	− 0.0142	− 0.0239	− 0.0133	− 0.0192
	(0.0131)	(0.0168)	(0.0133)	(0.0168)
OECD_pair	− 0.00754	− 0.00424	− 0.00709	− 0.00250
	(0.00887)	(0.00906)	(0.00885)	(0.00912)
EU_o	− 0.0115	− 0.0130	− 0.0108	− 0.0124
	(0.0180)	(0.0211)	(0.0180)	(0.0209)
EU_d	− 0.0271	− 0.0299	− 0.0296	− 0.0291
	(0.0217)	(0.0254)	(0.0219)	(0.0257)
EU_pair	0.0118	0.00693	0.0122	0.00859
	(0.00968)	(0.0104)	(0.00966)	(0.0102)
taxhaven_o	–	–	–	–
	–	–	–	–
taxhaven_d	–	–	–	–
	–	–	–	–
taxhaven_pair	0.00455	− 0.00254	0.00413	− 0.00578
	(0.00980)	(0.0106)	(0.00987)	(0.0106)
taxdividends_o	0.0375	− 0.0183	0.0420	− 0.00239
	(0.0624)	(0.0744)	(0.0629)	(0.0739)
taxdividends_d	− 0.0126	0.0278	− 0.0102	0.0262
	(0.0672)	(0.0810)	(0.0673)	(0.0824)
Time-fixed effects	Yes	Yes	Yes	Yes
Home-fixed effects	Yes	Yes	Yes	Yes
Host-fixed effects	Yes	Yes	Yes	Yes
Source-region-year fixed effects	No	Yes	No	Yes
Target-region-year fixed effects	No	Yes	No	Yes
Observations	879	837	879	837
R-squared	0.626	0.748	0.625	0.742

Robust standard errors in parentheses are clustered by country-pair ***p < 0.01; **p < 0.05; *p < 0.1

The dependent variable is the spread between the tax treaty withholding rate on portfolio and participation dividends in the host countries

## Footnotes

1 Tables 7 and 8 in the appendix look at the development over time by depicting the same constellations for the withholding tax rates for 2005 and 1980.

2 This approach has been applied to Figs. 2a, b and 3.

3 According to Gordon (1992), in the mid-1980s, the USA acted as if it were a Stackelberg leader by setting its corporate tax rate high, knowing that it would be in the interest of the other countries to match its rate. As the US economic dominance declined and with this also the role as a Stackelberg leader, the pressure for lower corporate tax rates may have increased.

4 Beside the newly signed double tax treaties, there were overall 166 (193) increases and 610 (215) decreases in the withholding tax rates on participation (portfolio) dividends in the existing double tax conventions for the period between 1950 and 2012.

5 The dataset covers the period between 1950 and 2012. However, domestic tax data for all countries are available only after 2004.

6 Another proxy for the extent to which countries are worried about tax avoidance and tax evasion is the tax morale variable from the World Values Survey (WVS 2015) that asks the question: “Do you justify cheating on taxes if you have the chance?” and takes the values between 1 (always) and 10 (never). The hypothesis is that countries, whose population is more likely to cheat on their taxes, may be worried about tax avoidance and tax evasion and therefore have an interest in a high tax treaty withholding rate on portfolio dividends in the host country. The results in Table 13 remain unchanged, and one can confirm the hypothesis that countries with population having a low tax morale are expected to have an interest in a high treaty withholding tax rate on portfolio dividends in the host country.

7 Two countries are defined as spatially connected if they share the same geographical region at the intermediate level according to the UN M49 standard (Petkova et al. 2020b).

8 Tables 5 and 6 present the results with a time lag of the spatial contagion variables with *n* = 2 years.

9 I look only at the one spatial lag, the target lag, as the source and target lag are identical for all cases in which the withholding tax rates included in the double tax treaties are the same in both directions. In other words, for every pair ij as well as ji, in which i is the source and j the target country, the WTRs are identical.

10 Tables 18 and 19 in the appendix reproduce Tables 5 and 6, but keep only new treaties as separate observations. Results remain robust.

11 In columns 4–6, not all multiple high-dimensional fixed effects (HDFE) are used, as they are not feasible with a probit estimation.

12 When it comes to double tax treaties, there are two main model conventions that countries could use as a guideline for establishing tax agreements—the Organization for Economic Cooperation and Development (OECD) Model Tax Convention on Income and on Capital and the United Nations Model Double Taxation Convention between Developed and Developing Countries (the UN Model Convention). By 1963, a full draft of the OECD Model Tax Convention was ready. It was the consolidation of four earlier drafts, the first one of which was published in 1958. Therefore, most people consider that the birth of the OECD Model Tax Convention was July 1, 1958 (OECD Observer No. 269, October 2008). The draft United Nations Model Convention was reviewed by the Group of Experts in Geneva in 1979, and the final text of the Convention was adopted (United Nations 1980, E.80. XVI.3).

13 It should also be noted that the threshold to qualify for foreign direct investment (FDI) and therefore for the lower withholding tax rate on participation dividends, as opposed to portfolio investment and the higher withholding tax rate on portfolio dividends, is lower in the UN Model Convention than in the OECD Model Tax Convention—10% versus 25% of the capital of the company paying the dividends (Lennard 2008).

14 For a reproduction of Table 5 in the same way, look at Table 17 in the appendix.
